# Evaluation of Soil Antagonism against the White Root Rot Fungus *Rosellinia necatrix* and Pathogen Mycosphere Communities in Biochar-amended Soil

**DOI:** 10.1264/jsme2.ME24060

**Published:** 2024-12-20

**Authors:** Yong Guo, Sachie Horii, Satoko Kanematsu

**Affiliations:** 1 Institute for Plant Protection, National Agriculture and Food Research Organization (NARO), Tsukuba 305–8605, Japan; 2 Institure of Fruit Tree and Tea Science, National Agriculture and Food Research Organization (NARO), Tsukuba 305–8605, Japan

**Keywords:** *Rosellinia necatrix*, soil antagonism, Japanese pear orchard, pruned branch biochar, mycosphere microbiome

## Abstract

White root rot disease caused by *Rosellinia necatrix* is a growing issue in orchards, and biochar pyrolyzed from the pruned branch residues of fruit trees has potential as a soil amendment agent with a number of benefits, such as long-term carbon sequestration. However, the effects of pruned branch biochar on white root rot disease remain unclear. Therefore, we compared direct antagonism against *R. necatrix* between soils with and without pruned pear branch biochar using a toothpick method and then linked soil physicochemical properties and microbial communities with soil antagonism. The results obtained showed that soil antagonism against the pathogen, that is, the extinction zone of *R. necatrix* in mycelial toothpicks, decreased in soils amended with 20% (v/v) pruned branch biochar. Soil pH was neutralized and aeration was promoted by the biochar amendment, which may be favorable for pathogen growth. An investigation of microbial communities surrounding *R. necatrix* mycelia indicated that antagonistic fungi affiliated with *Chaetomiaceae* and *Trichoderma* were selectively excluded from the mycosphere community in biochar-amended soil. Therefore, the enrichment of these indigenous antagonistic fungi may be important for controlling *R. necatrix*. Based on the present results, we do not recommend the application of pruned branch biochar to the soil area associated with the roots of fruit trees in order to avoid increasing the risk of white root rot in orchards.

White root rot caused by the ascomycete fungus *Rosellinia necatrix* is one of the most serious soil-borne diseases threatening wood crops in temperate and subtropical areas worldwide (Pérez-Jiménez, 2006; Pliego *et al.*, 2012). As a pathogenic agent, *R. necatrix* infects and necrotizes the roots of economically important plants, such as apples, pears, peaches, grapes, avocados, and olives (Sztejnberg and Madar, 1980). Host attacks by *R. necatrix* depend on soil temperature, oxygen, the water balance, organic matter content, pH, and soil microbiome (Pliego *et al.*, 2012; Carlucci *et al.*, 2013); therefore, changes in these soil features have a reasonable impact on disease occurrence.

Biochar is a carbon-enriched solid produced by pyrolyzing biomass materials under oxygen-deficient conditions at high temperatures (Lehmann *et al.*, 2006). As a soil amendment agent, biochar has potential for long-term carbon sequestration, greenhouse gas mitigation, and waste management (Lehmann *et al.*, 2006; Lehmann, 2007; He *et al.*, 2017). With respect to control strategies for plant diseases, a growing number of studies have revealed that the application of biochar effectively suppresses phytopathogens through various mechanisms, such as changing soil physicochemical properties, inducing systemic plant defenses, adsorbing toxins produced by pathogens, and reshaping the soil microbiome (Poveda *et al.*, 2021). Moreover, suppressive effects have mainly been reported on soil-borne diseases caused by the bacterium *Ralstonia solanacearum* and by the fungi *Rhizoctonia solani* and *Fusarium* species (Poveda *et al.*, 2021; Arshad *et al.*, 2024). In addition, the effectiveness of this suppression appears to differ with the crop type. A global meta-ana­lysis showed that biochar effectively decreased disease severity in vegetables, berries, and tobacco; however, this suppressive effect was limited in cereal grains and perennial trees (Yang *et al.*, 2022). Further studies are required to examine the biochar-mediated control of more diverse phytopathogens, particularly those that are harmful to perennial wood crops.

The Japanese pear (*Pyrus pyrifolia* Nakai), one of the most widely cultivated fruit trees in Japan, is typically pruned during the winter to encourage more fruiting buds and reduce bud height (Bound, 2022). The return of pruned branch residues to orchard soil is not recommended because of the increased risk of plant disease, and usual disposal by open burning results in the general emission of greenhouse gases and an adverse environmental impact. As an alternative to open burning, soil amendment with biochar pyrolyzed from pruned branch residues has the potential to reduce CO_2_ and N_2_O emissions from pear orchards (Oo *et al.*, 2018), suggesting a novel carbon-neutral strategy suitable for wood crops. However, some wood waste biochars increase the severity of damping-off caused by *R. solani* in various crops (Copley *et al.*, 2015). Therefore, proper risk management and careful evaluations are essential when considering biochar use (Godlewska *et al.*, 2021; Tan and Yu, 2024).

Due to the serious perniciousness of white root rot disease in pear cultivation, the present study exami­ned the effects of pruned pear branch biochar on the pathogenic fungus *R. necatrix*. A useful evaluation method using *R. necatrix*-colonized toothpicks (Takahashi and Nakamura, 2020) was employed to compare soil antagonism against the pathogen in soils with and without the application of pruned branch biochar. The physicochemical properties of soil and the microbiome surrounding mycelia of the pathogen were also investigated to identify the abiotic and biotic factors affecting soil antagonism against *R. necatrix*. The results obtained herein provide insights into the development of information-based biocontrol strategies for soil-borne diseases, which are essential for the application of pruned branch biochar in orchards.

## Materials and Methods

### Study sites, soil sampling, and preparation

Four orchards with different treatments were selected to evaluate the effects of the biochar amendment on soil antagonism against the white root rot fungus *R. necatrix* (*Rn*). Three of these orchards, Tsukuba-A, Tsukuba-B, and Tsukuba-C, located in the National Agriculture and Food Research Organization (NARO) in Tsukuba, Japan, were continuously planted with the Japanese pear ‘Kosui’ for more than 10 years. Undergrowth vegetation in Tsukuba-A and Tsukuba-B was managed automatically without any herbicide and pruned by a robot lawn mower, whereas that in Tsukuba-C was exterminated by the periodic spraying of herbicides. In Tsukuba-A and half of Tsukuba-B (Tsukuba-B1), the undergrowth was dominated by low gramineous grasses (height <5‍ ‍cm), whereas the other half of Tsukuba-B (Tsukuba-B2) was mainly covered by high Chenopodiaceae grasses (height >20‍ ‍cm). An orchard located in the Chiba Prefectural Agriculture and Forestry Research Center in Chiba, Japan, named ‘Chiba’, was continuously planted with Japanese pear ‘Akizuki’, and the undergrowth in this orchard was managed by the periodic spraying of herbicides. The soil in all four orchards was classified as Andosol.

Six soil samples were collected from the four orchards and prepared to evaluate antagonism against *R. necatrix* according to the procedure reported by Takahashi and Nakamura (2020). Briefly, soil between depths of 5 and 30‍ ‍cm was collected from a 20×20‍ ‍cm square that was 50‍ ‍cm away from the pear tree trunk. Soil samples S1 and S2 were collected from Tsukuba-A in May 2023 and July 2023, respectively. Samples S3, S4, and S5 were collected from Tsukuba-B1, Tsukuba-B2, and Tsukuba-C, respectively, in October 2023. Sample S6 was collected from the Chiba Orchard in August 2023. All soil samples were sieved through a 4-mm mesh for use. The biochar used in this study was prepared by pyrolyzing the pruned branch of Japanese pear in a smokeless carbonizer (MOKI) at a temperature of approximately 600°C, as described in a previous study (Oo *et al.*, 2018). Biochar (size–2–4‍ ‍mm) was sieved and mixed thoroughly with soil samples at a volume ratio of 20% (equivalent to a weight ratio range of 5–6%) for the evaluation of antagonism.

### Evaluation of soil antagonism against R. necatrix

In the present study, *R. necatrix* strains W97 (MAFF625116) and W563 (MAFF645027) were used to evaluate soil antagonism against *R. necatrix*. The toothpick method was performed as described by Takahashi and Nakamura (2020). Each tested strain was preincubated in a polycarbonate box (CUL-JAR300; AGC TECHNO GLASS) containing 50‍ ‍mL of potato dextrose agar (PDA) (Difco) at 23°C for 3 days; another box with 25 holes (2.5‍ ‍mm in diameter) on the bottom was then stacked onto the first box. Birchwood toothpicks (65‍ ‍mm in length) autoclaved in distilled water were inserted into the holes of the upper box to reach the fungal colonies in the lower box. After covering the upper box with a lid, this set was incubated at 23°C for 2–3‍ ‍weeks until the toothpicks were fully colonized by mycelia. The upper box with toothpicks was recovered, and 0–10‍ ‍mm of the tips of the toothpicks were immersed in 45°C water for 30‍ ‍min to kill *R. necatrix* inhabiting the tips. The upper box was then stacked onto a new box containing 160‍ ‍mL of the tested soil to place 0–30‍ ‍mm of the tips of the toothpicks into the soil. This set was incubated at 23°C for 4‍ ‍weeks to measure the length of the extinction zone of *R. necatrix* mycelia, *i.e.*, the distance from the toothpick tip to the mycelial margin (Supplementary Fig. S1), under a stereomicroscope (Leica). The length of the extinction zone was measured for 16 toothpicks in the periphery, and the mean value of the 16 toothpicks was calculated for each device. A single experiment was performed for each soil sample in triplicate, *i.e.*, using three sets of toothpick devices. The mean value of three devices was calculated as the result of soil antagonism. Soil without biochar, that is, biochar-free soil, was used as the control (CS).

### Assessing the severity of white root rot in biochar-amended soils (BS) in pots

*R. necatrix* attacks more than 170 hosts of both herbaceous and woody plants (Pliego *et al.*, 2012), and some leguminous and rosaceous plants have been used to estimate the virulence of this fungus (Uetake *et al.*, 2001; Kanematsu *et al.*, 2004). Therefore, to further assess the disease severity of white root rot in BS, *R. necatrix* strains W97 and W563 were inoculated into mung (*Vigna radiata*) and apple (*Malus prunifolia* var. ringo) plants in pots, according to Kanematsu (2014) with minor modifications. Soil between depths of 5 and 30‍ ‍cm was collected from a fallow orchard in NARO in Tsukuba and mixed thoroughly with 20% (v/v) pear branch biochar. The inoculum of *R. necatrix* was prepared using wheat grains according to Sawant *et al.* (2023) with minor modifications. Briefly, 200‍ ‍g of wheat grain was soaked in 300‍ ‍mL of distilled water for 5 h, autoclaved, and inoculated with five pieces (7‍ ‍mm in diameter) of the 7-day-old PDA culture of *R. necatrix*. After incubating at 23°C for 2‍ ‍weeks, the fungus grew uniformly, fully covering the wheat grains. The inoculum used for apples was prepared according to the procedure described by Kanematsu *et al.* (2004). One-year-old shoots of Japanese pear (7–10‍ ‍mm in diameter) were cut into 20-mm-long fragments, soaked in distilled water, and autoclaved. Approximately 10–12 of the autoclaved fragments were placed on a PDA dish with the 7-day growing pathogen and incubated at 23°C for 4‍ ‍weeks, until shoot fragments were entirely covered by *R. necatrix* mycelia.

Mung seeds were germinated at 20°C for 2 days in a Petri dish with a piece of wet filter paper. Two germinated seeds were sown in BS in the center of a 300-mL pot, whereas a 1-year-old apple plant (approximately 10‍ ‍mm in diameter) was transplanted into BS in a 1,500-mL pot. Seven mung pots and six apple pots were prepared and cultivated in a 25°C phytotron for 2‍ ‍weeks and 4‍ ‍weeks, respectively, until the inoculation with *R. necatrix*. Regarding the inoculation, two grains of *R. necatrix*-colonized wheat seeds were placed for attachment to the root at a depth of 1‍ ‍cm for each mung seedling, whereas two pieces of *R. necatrix*-colonized shoot fragments were inoculated on the root at a depth of 7‍ ‍cm for each apple plant. The pots were cultivated in the 25°C phytotron. Disease severity on mung and apple plants was estimated at 11 days post-inoculation (dpi) and 38 dpi, respectively, and rated as follows: 0, healthy; 1, wilting of <50% leaves; 2, wilting of ≥50% leaves; 3, entirely withered and dead. Inoculation experiments were repeated twice for each plant.

### Soil physicochemical ana­lysis

To measure soil physicochemical properties, 50 to 500‍ ‍g of each soil mixed thoroughly with 20% (v/v) of the pear branch biochar was packed in a sterile plastic bag and stored at 4°C until the ana­lysis. Total carbon (TC) and total nitrogen (TN) were assessed using an NC analyzer (SUMIGRAPH NC-220F; Sumika Chemical Analysis Service). The volumetric water content was analyzed manually by drying the soil sample in an oven set at 105°C overnight. A slurry comprising a 1:2.5 mass ratio of the sample to deionized water was used to evaluate soil pH. CS was analyzed as a control.

### R. necatrix mycosphere collection, DNA extraction, and meta-amplicon sequencing

Mycospheres of *R. necatrix* (*Rn*-M), which consist of the microbiome assembled around the mycelia of *R. necatrix*, were recovered from mycelial toothpicks after the incubation in the tested soil, as shown in Supplementary Fig. S2. In brief, after an incubation at 23°C for 4‍ ‍weeks, mycelial toothpicks were pulled out and loose soil particles were removed by shaking the device. Soil particles that adhered tightly to mycelial toothpicks were stripped off with sterile forceps. All stripped soils from the same box were mixed in a sterile plastic bag and stored at –20°C until DNA extraction. Since the strongest antagonism against *R. necatrix* was observed in S1 soil, only *Rn*-M microbial communities associated with S1 soil were subsequently analyzed. Additionally, two boxes without mycelial toothpicks, one containing biochar-amended S1-soil and the other containing biochar-free S1-soil, were incubated during the same period of the soil antagonism evaluation, and the microbial communities in the two boxes were analyzed as negative controls for *Rn*-M. Therefore, we ultimately analyzed 14 samples: six for *Rn*-M in BS (W97-M in BS×3 and W563-M in BS×3), six for *Rn*-M in CS (W97-M in CS×3 and W563-M in CS×3), one for BS without exogenous *R. necatrix* (*Rn*-free BS×1), and one for CS without exogenous *R. necatrix* (*Rn*-free CS×1).

In each sample, total DNA was extracted from 0.5‍ ‍g of soil using a NucleoSpin Soil Kit (Macherey-Nagel) according to the manufacturer’s instructions. The concentration and purity of genomic DNA were measured using a Qubit^®^ 2.0 fluorometer (Life Technologies) with a dsDNA HS Assay kit (Life Technologies) and NanoDrop 2000 spectrophotometry (NanoDrop Technologies). The primer set of ITS3-2024F (5′-GCATCGATGAAGAACGCAGC-3′) and ITS4-2409R (5′-TCCTCCGCTTATTGATATGC-3′) was used to target the fungal ITS2 region (White *et al.*, 1990). Primers 515F (5′-GTGCCAGCMGCCGCGGTAA-3′) and 806R (5′-GGACTACHVGGGTWTCTAAT-3′) were used to amplify the V4 hypervariable regions of the prokaryotic 16S rRNA gene (Caporaso *et al.*, 2011). Library construction and amplicon sequencing were conducted using an Illumina NovaSeq 6000 platform by Novogene Japan (Novogene). Sequencing was performed to generate >50,000 and >100,000 paired-end tags (2×250 bp) per sample for the fungal ITS2 and prokaryotic 16S rRNA V4 regions, respectively.

### Bioinformatics ana­lyses

After removing the adaptors and primer sequences, raw reads were assembled for each sample according to a unique barcode using QIIME 2 (Bolyen *et al.*, 2019). Paired-end reads for each sample were merged using FLASH v2.2.00 with default settings, except for a quality cut-off of 30 (Magoč and Salzberg, 2011). The sequences obtained for all samples were processed using the Mothur pipeline reported by Guo *et al.* (2014) with minor modifications. Briefly, sequences containing ambiguous bases and those shorter than 200 bp or homopolymer lengths longer than 8 bp were discarded. The qualified sequences were denoised using the ‘pre.cluster’ command, followed by the removal of chimera using the ‘chimera.vsearch’ command in Mothur. The remaining sequences were assigned to operational taxonomic units (OTUs) with a 97% identity threshold using the OptiClust clustering method (Westcott and Schloss, 2017). Fungal OTUs were classified using UNITE database v9 (Kõljalg *et al.*, 2013), and prokaryotic OTUs were annotated against RDP database v18 (Wang *et al.*, 2007) using the RDP classifier in Mothur with an 80% confidence threshold (Schloss *et al.*, 2009). The fungal OTUs assigned to *R. necatrix* were trimmed, and the remaining fungal composition was defined as the fungal community of *R. necatrix* mycospheres.

We assessed the diversity and structure of the microbial community using 1,000-time-repeated random subsampling at the size of the smallest fungal and prokaryotic libraries. The OTU count (*i.e.*, observed species number), Shannon–Wiener index, and Chao1 richness were calculated at a 0.03 cut-off level to assess fungal and prokaryotic diversities. A pairwise distance matrix was established for fungal and prokaryotic communities using Bray–Curtis dissimilarities, and a principal coordinate ana­lysis (PCoA) was performed on distance matrices to visualize the communities in two-dimensional scattergrams. An ana­lysis of mole­cular variance (AMOVA) was conducted to estimate significant differences in the community structure of *Rn*-M in BS and CS. Additionally, the linear discriminant ana­lysis effect size (LEfSe) was used to identify differentially abundant OTUs between *Rn-*M in BS and CS (Segata *et al.*, 2011). The threshold used for the logarithmic linear discriminant ana­lysis (LDA) score was 2.0, and significance was set at *P*<0.05 using the Wilcoxon test.

### Statistical ana­lysis

The Student’s *t*-test was performed to evaluate significant differences in soil physicochemical properties, except for the water content, length of the *R. necatrix* extinction zone, and microbial diversity indices, whereas Fisher’s exact test was used to examine significant differences in the disease score of white root rot in the pots. The water content was represented using a percentage and, thus, the significance of differences in the water content between soils with and without biochar was exami­ned by the Mann–Whitney U test. Significance was defined as *P*<0.05. Pearson’s and Spearman’s correlation coefficients were used to evaluate linear and monotonic relationships between the length of the *R. necatrix* extinction zone and soil physicochemical properties as well as the relationships between the extinction zone and *Rn*-M microbial indicators (OTU count, Shannon–Wiener index, Chao1 richness, PCoA1, and PCoA2). Pearson’s correlation coefficients were also calculated to identify the major fungal and prokaryotic OTUs in *Rn*-M correlating with the length of the extinction zone, that is, soil antagonism against *R. necatrix*. All statistical ana­lyses were performed in R platform v4.3.0 (available at https://www.r-project.org/), and correlation ana­lyses were conducted using the R packages ‘Hmics v5.1-2’ and ‘Matrix v1.5-4’.

### Sequence accession numbers

All raw read data were deposited in the DDBJ Sequence Read Archive (DRA) database under the accession numbers DRA018799 and DRA018800 for prokaryotic 16SV4 and fungal ITS2 reads, respectively. These data are available upon reasonable request.

## Results

### Antagonism against *R. necatrix* in BS

Six soil samples collected from four Japanese pear orchards with different locations, sampling times, management, and undergrowth vegetation types were evaluated for the effects of the biochar amendment on soil antagonism against the phytopathogenic fungus *R. necatrix* using two well-studied stains, W97 and W563, in Japan. The results obtained are summarized in Table 1. Although the evaluation showed a broad range of antagonism against *R. necatrix* for the different samples, the *R. necatrix* extinction zone was shorter in all tested BS than in the control (*i.e.*, the corresponding CS). A significant decrease in the *R. necatrix* extinction zone was observed for eight pairs of comparisons (the Student’s *t*-test, *P*<0.05), accounting for 66.7% of observations using the toothpick method. These results clearly indicate that soil antagonism against *R. necatrix* decreased when 20% (v/v) of pruned branch biochar was mixed into the soil.

### Disease severity of white root rot in BS

According to expectations from observations using the toothpick method, the disease scores of both *Rn*-inoculated mung and apple plants were higher in pots with BS (Table 2). Regarding *R. necatrix* W97, a significant difference in disease severity between pots with and without the biochar amendment was observed for both plant species (Fisher’s exact test, *P*<0.05). These results further revealed that white root rot was stimulated when 20% (v/v) pruned branch biochar was mixed into the soil.

### Relationships between soil physicochemical properties and antagonism against *R. necatrix*

TC, TN, the water content, and pH were assessed in BS and CS to evaluate antagonism against *R. necatrix*. The amendment with 20% (v/v) biochar significantly increased soil TC and pH (the Student’s *t*-test, *P*<0.01) (Table 3). Although TN also significantly differed between soils with and without biochar, the range of the change in TN was small and inconsistent (Table 3). The relationships between soil physicochemical properties and antagonism against *R. necatrix* were further estimated (data not shown); however, only pH negatively correlated with the length of the *Rn* extinction zone (Pearson’s R=–0.48, *P*=0.02; Spearman’s R=–0.51, *P*=0.01).

### Microbial community diversity and structure of *R. necatrix* mycosphere in biochar

Due to the relatively high antagonism estimated for S1 soil, an investigation of the microbial community in the soil was expected to reveal the biotic indicators responsible for the antagonistic potential. Regarding the closest association with *R. necatrix*, soil microbial communities around the mycelial toothpicks, that is, *Rn*-M communities, were analyzed using a culture-independent approach. Fungal and prokaryotic OTU counts, Chao1 richness, and Shanno–Wiener indices are summarized in Table 4. Approximately 300–390 fungal and 3,400–4,800 prokaryotic OTUs were detected in *Rn*-M communities. In comparisons with *Rn*-free communities, only fungal OTU counts were markedly lower. Similar results were observed in Chao 1 richness and Shannon–Wiener indices. No significant difference in microbial diversity was observed between *Rn*-M communities with and without biochar, expect for the fungal Shannon–Wiener index of the W97 mycosphere community, which was significantly higher in BS (the Student’s *t*-test, *P*<0.01).

**Fig. 1.**
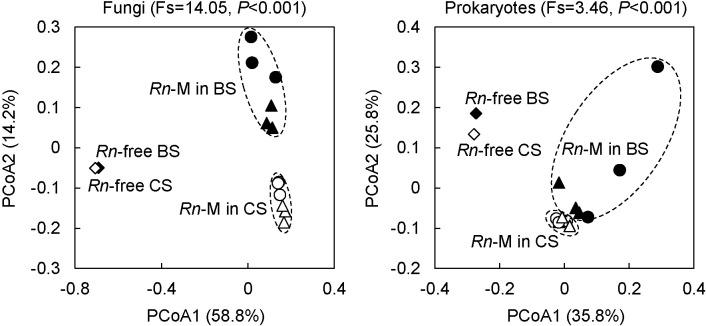
Principle coordinate ana­lysis (PCoA) of fungal (left) and prokaryotic (right) community structures in the mycosphere of *Rosellinia necatrix* (*Rn*-M) in soils with and without biochar. Two-dimensional plots are generated according to Bray–Curtis dissimilarities based on 97% identity operational taxonomic units. An ana­lysis of mole­cular variance (AMOVA) was performed to estimate significant differences in the community structure of *Rn*-M between soils with and without biochar. A non-parametric fixation index (Fs) >1 indicates that the compared communities are significantly different. ▲, W97-M in BS; ●, W563-M in BS; ◆, *Rn*-free BS; △, W97-M in CS; ○, W563-M in CS; ◇, Rn-free CS.

As shown by the PCoA plots in Fig. 1, the fungal communities of *Rn*-M in BS were clearly separate from those of *Rn*-M in CS and *Rn*-free soils (AMOVA, Fs=14.05, *P*<0.001). Similar results were observed for prokaryotic communities; however, the difference (AMOVA, Fs=3.46, *P*<0.001) was less than that in fungal communities. In addition, the prokaryotic community in the W563-mycoshere highly varied among individual samples, suggesting that specific prokaryotes occasionally inhabited the W563 mycosphere.

### Taxonomic composition of *Rn*-M communities

**Fig. 2.**
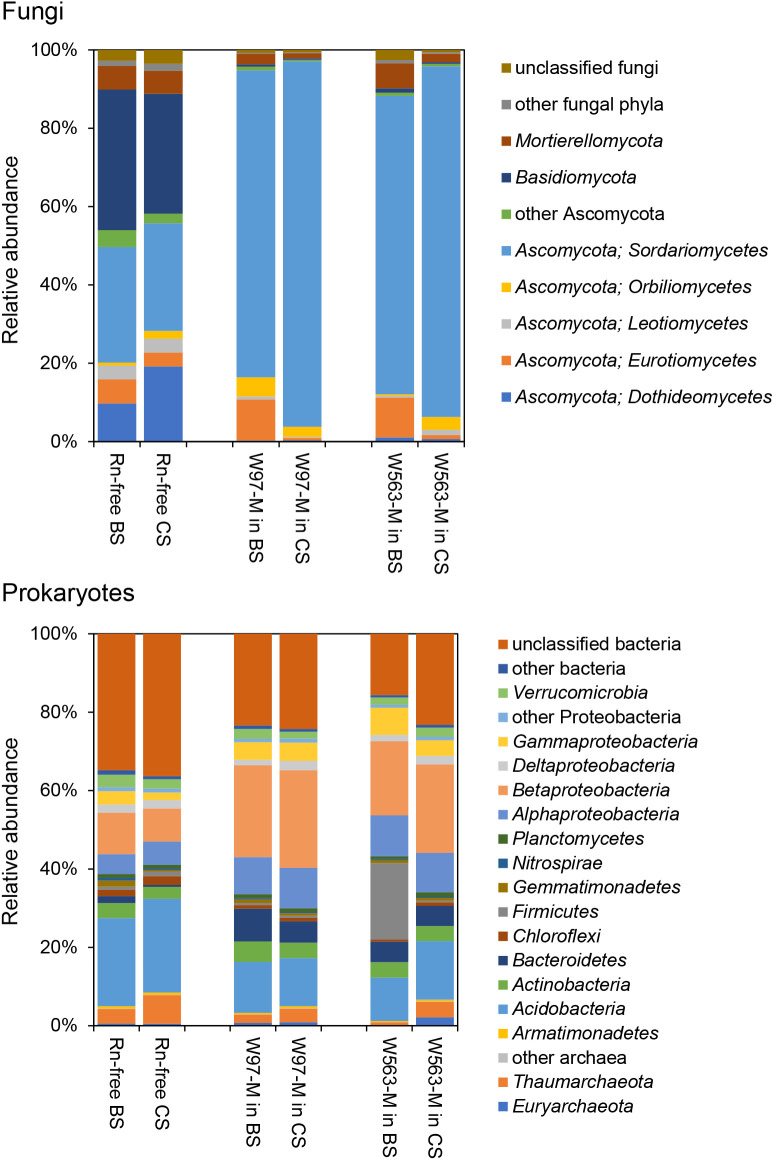
The relative abundance of fungal (upper) and prokaryotic (lower) taxa in the mycosphere community of *Rosellinia necatrix* in soils with and without biochar. Bar plots of relative abundance for *R. necatrix* (*Rn*) mycosphere communities (W97-M and W563-M) in soils with and without biochar represent the mean values of taxonomic units of triplicate measurements, and those for *Rn*-free communities represent a single measurement.

The taxonomic compositions of 846,875 fungal ITS sequences and 2,030,983 prokaryotic 16S-V4 sequences across the 14 samples were analyzed. In the fungal ITS ana­lysis, a single OTU classified as *R. necatrix* was the most abundant fungus in samples, accounting for 9 to 57% of all ITS reads generated from samples that adhered to the mycelial toothpicks (data not shown); however, it was not detected in *Rn*-free samples. After removing the *R. necatrix* OTU, the remaining fungal OTUs were designed as fungal communities in *Rn*-M. The fungal communities in *Rn*-M were dominated by a single phylum, Asocomycota, with a major class of Sordariomycetes (Fig. 2). In comparisons with the fungal communities of *Rn*-M in CS, the Ascomycota class Eurotiomycetes was more abundant in *Rn*-M in BS (Fig. 2). In contrast, the prokaryotic communities of *Rn*-M mainly comprised five bacterial phyla: *Proteobacteria* (major classes of *Alpha-*, *Beta-*, *Gamma-*, and *Delta-Proteobacteria*), *Acidobacteria*, *Actinobacteria*, *Bacteroidetes*, and *Verrucomicrobia*, as well as two archaeal phyla, *Thaumachaeota* and *Euryarchaeota* (Fig. 2). Moreover, the bacterial phylum *Firmicutes* was more abundant in W563-M in BS (Fig. 2). However, this percentage was mainly derived from a single OTU belonging to the genus *Listeria* in two individual samples of W563-M in BS (data not shown). Additionally, unclassified bacteria and taxa of *Acidobacteria*, *Dothideomycetes*, and *Basidiomycota* were more abundant in *Rn*-free soils (Fig. 2).

**Fig. 3.**
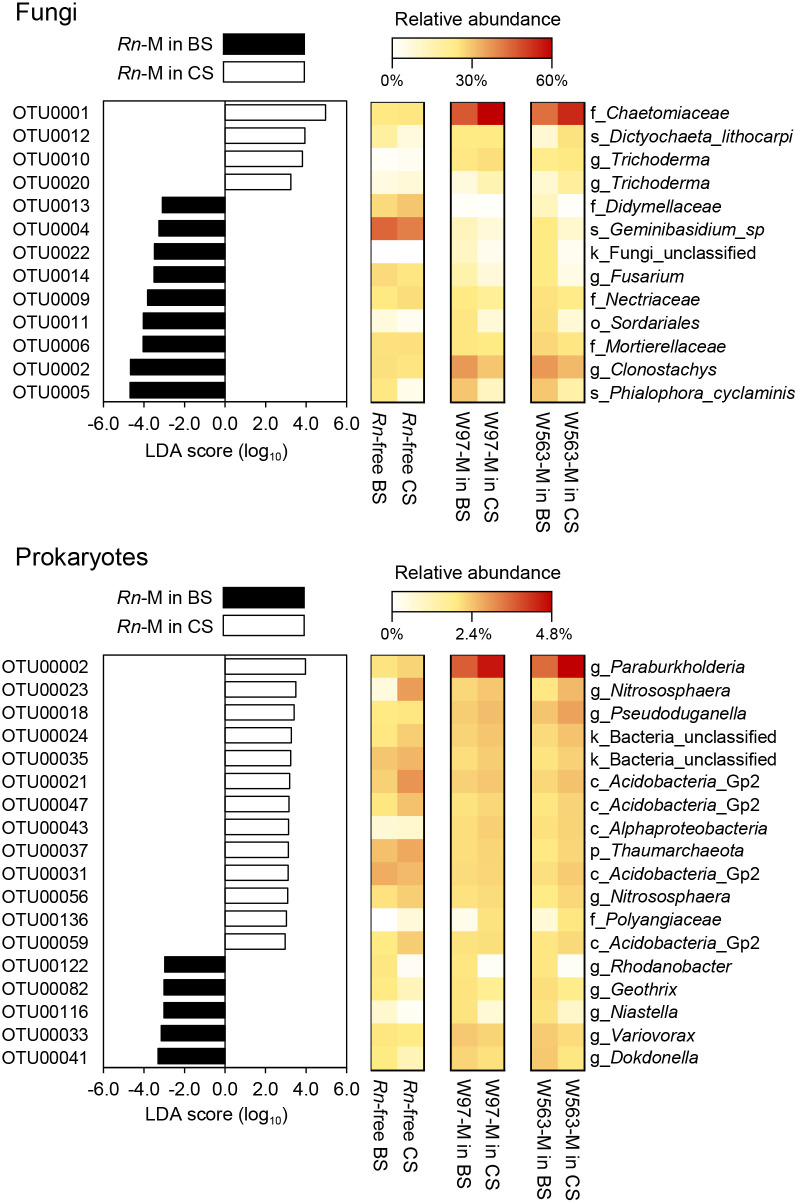
Histograms of logarithmic linear discriminant ana­lysis (LDA) scores calculated for differentially abundant fungal (upper) and prokaryotic (lower) OTUs in the mycosphere community of *Rosellinia necatrix* (*Rn*-M) between soils with and without biochar. Linear discriminant ana­lysis effect size (LEfSe) was performed with the datasets of fungal and prokaryotic OTUs in *Rn*-M communities, *i.e.*, six *Rn*-M communities in soils with biochar and six without biochar. Significantly abundant OTUs with LDA scores >3.0 are shown in the histograms. Heatmaps show the relative abundance of each selected OTU in different soil or mycosphere samples. The heatmaps for *Rn*-M communities (W97-M and W563-M) in soils with and without biochar represent the mean values of triplicate measurements, and those for *Rn*-free communities represent a single measurement.

We used LEfSe to detect the relationship between fungal and prokaryotic OTUs in *Rn*-M with BS and CS. Twenty-four fungal OTUs and 66 prokaryotic OTUs were more abundant in *Rn*-M in BS, whereas 7 fungal OTUs and 51 prokaryotic OTUs were more prevalent in *Rn*-M in CS (Table S1). Regarding major OTUs with an absolute LDA score >3 (Fig. 3), 9 fungal OTUs and 5 prokaryotic OTUs were more abundant in *Rn*-M in BS, whereas 4 fungal OTUs and 13 prokaryotic OTUs were more abundant in *Rn*-M in‍ ‍CS. The fungal OTUs more abundant in *Rn*-M in BS were identified as the taxa of *Phialophora cyclaminis*, *Clonostachys*, *Fusarium*, *Geminibasidium*, *Mortierellaceae*, *Nectriaceae*, and *Didymellaceae*, whereas those more abundant in *Rn*-M in CS were *Dictychaeta lithocarpi*, *Trichoderma*, and *Chaetomiaceae*. Moreover, the prokaryotic OTUs that were more abundant in *Rn*-M in BS were identified as the taxa‍ ‍*Dokdonella*, *Variovorax*, *Niastella*, *Geothrix*, and *Rhodanobacter*, whereas those more abundant in *Rn*-M in‍ ‍CS were *Paraburkholderia*, *Nitrosophaera*, *Pseudoduganella*, *Polyangiaceae*, *Acidobacteria* Gp2, and unclassified bacteria.

### Relationships between microbial indicators and antagonism against *R. necatrix*

Microbial indicators involved in diversity and community structures were estimated for linear and monotonic correlations with the soil antagonism of *R. necatrix* (Table 5). Notably, fungal Shannon–Wiener diversity (Pearson’s R=–‍0.66, *P*=0.020; Spearman’s R=–0.66, *P*=0.018) and fungal PCoA2 (Pearson’s R=–‍0.63, *P*=0.028; Spearman’s R=–‍0.62, *P*=0.033) negatively correlated with antagonism against *R. necatrix*, whereas fungal PCoA1 (Pearson’s R=0.83, *P*=0.001; Spearman’s R=0.78, *P*=0.003) positively correlated with this antagonism. In addition, prokaryotic PCoA1 (Pearson’s R=–0.60, *P*=0.038) and PCoA2 (Spearman’s R=–0.61, *P*=0.036) showed negative linear and monotonic relationships with antagonism, respectively.

**Fig. 4.**
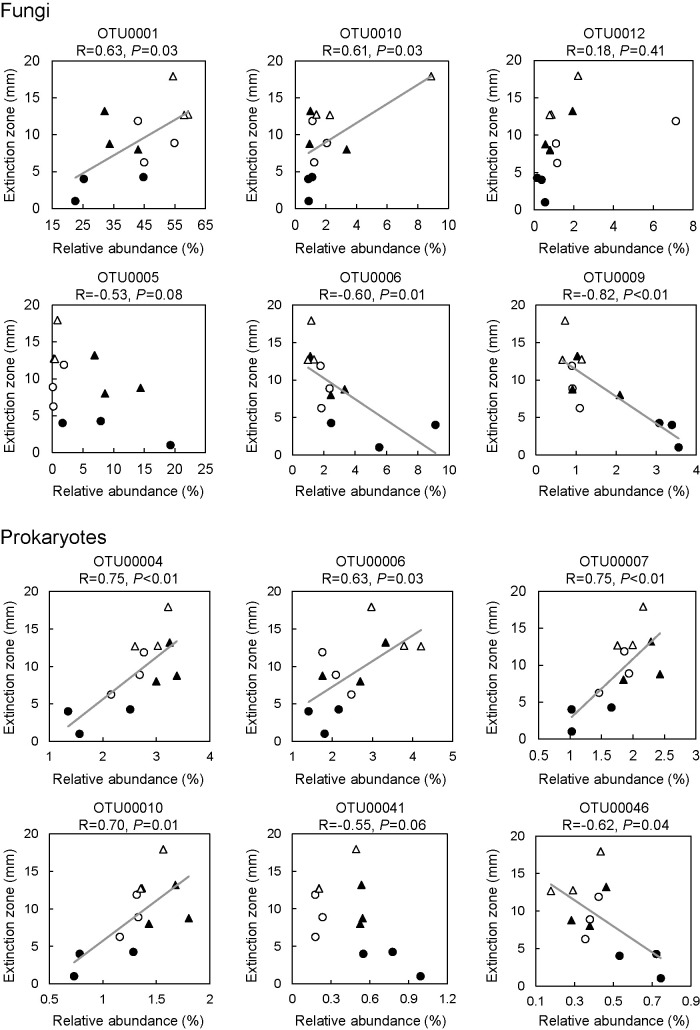
Linear regression relationship between specific OTUs and the extinction zone of *Rosellinia necatrix* in mycelial toothpicks. Correlations (*P*<0.05) are indicated with a linear regression curve in the plot. The taxonomy of each selected fungal OTU is identified as follows: OTU0001, *Chaetomiaceae*; OTU0005, *Phialophora cyclaminis*; OTU0006, *Mortierellaceae*; OTU0009, *Nectriaceae*; OTU0010, *Trichoderma*; OTU0012, *Dictyochaeta lithocarpi*. The taxonomy of each selected prokaryotic OTU is identified as follows: OTU00004, unclassified bacterium; OTU00006, Nitrosomonadales; OTU00007, unclassified bacterium; OTU00010, unclassified bacterium; OTU00041, *Dokdonella*; OTU00046, *Rhodanobacteraceae*. ▲, W97-M in BS; ●, W563-M in BS; △, W97-M in CS; ○, W563-M in CS.

Furthermore, we used Pearson’s linear regression to evaluate the relationship between the length of the *Rn* extinction zone and major bacterial and fungal OTUs in *Rn*-M (Fig. 4 and Table S2). The relative abundance of fungal OTU0001 (*Chaetomiaceae*) and –0010 (*Trichoderma*) and those of prokaryotic OTU00004 (unclassified bacterium), –00006 (Nitrosomonadales), –00007 (unclassified bacterium), and –‍00010 (unclassified bacterium) positively correlated (*P*<0.05) with antagonism against *R. necatrix*. Conversely, the relative abundance of fungal OTU0006 (*Mortierellaceae*) and –‍0009 (*Nectriaceae*), and that of prokaryotic OTU00046 (*Rhodanobacteraceae*) negatively correlated (*P*<0.05) with the *Rn* extinction zone.

## Discussion

The application of biochar to soils suppresses soil-borne diseases caused by fungal phytopathogens, such as *Fusarium* spp. and *R. solani* (Poveda *et al.*, 2021; Iacomino *et al.*, 2022; Yang *et al.*, 2022; Arshad *et al.*, 2024); however, the effects of biochar on the control of the white root rot fungus *R. necatrix* remain unclear. In the present study, we exami­ned soil antagonism against *R. necatrix* in BS. By using the toothpick method, a simple assessment approach (Takahashi and Nakamura, 2020), we found that the extinction zone of *R. necatrix* in the mycelial toothpick, *i.e.*, soil antagonism against *R. necatrix*, significantly decreased when 20% (v/v) of pruned branch biochar was amended in orchard soils. This result was further confirmed by the increased disease severity of white root rot in mung and apple plants cultivated in BS. Therefore, in contrast to previous findings, the present results revealed a conducive effect of the biochar amendment on white root rot disease.

The physicochemical features of soil were further analyzed to clarify the abiotic factors driving soil antagonism against *R. necatrix*. The biochar amendment significantly increased TC, TN, and pH in soil, whereas only pH negatively correlated with antagonism. Under natural conditions, *R. necatrix* infects host plants in soils with pH between 6 and 8 (Pérez-Jiménez, 2006). In the present study, the biochar amendment neutralized soil pH from 5.2~6.0 to 6.6~7.6, appearing to create a favorable acid-base environment for the pathogen. In addition, low soil aeration was unfavorable for the disease occurrence of *R. necatrix* (Pliego *et al.*, 2012), whereas a previous study showed that a 5–10% biochar amendment increased soil aeration (Case *et al.*, 2012). Therefore, a biochar amendment may improve gas-phase conditions, namely, the availability of oxygen in soil, to the benefit of *R. necatrix* attacks.

Microbial competition between pathogens and surrounding soil microbes is important for disease development (Snelders *et al.*, 2020), whereas antagonistic bacteria and fungi are key players in the suppression of *R. necatrix* (Arjona-López *et al.*, 2019b; Takahashi *et al.*, 2020). In the present study, we characterized the microbial communities of *R. necatrix* mycospheres associated with the biochar amendment. The results obtained showed no significant differences in the microbial diversity or phylum composition of *R. necatrix* mycosphere communities. Sordariomycetes dominated the fungal communities of *R. necatrix* mycosphere in soils with and without biochar, whereas prokaryotic communities comprised *Proteobacteria*, *Acidobacteria*, *Actinobacteria*, *Bacteroidetes*, *Verrucomicrobia*, *Thaumachaeota*, *Euryarchaeota*, and unclassified bacteria. This phylum-based taxonomic composition is similar to that of two other soil-borne disease fungi, *Fusarium oxysporum* (Sun *et al.*, 2021; Thomas and Antony-Babu, 2024) and *Verticillium dahliae* (Batista *et al.*, 2024), suggesting that soil-borne pathogenic fungi recruit a similar subset from the soil microbiome to form a pathobiome. Nevertheless, fungal and prokaryotic communities in *R. necatrix* mycosphere varied in structure at the OTU level (≥97% identity) in relation to the biochar amendment.

LEfSe revealed specific microbial groups associated with *R. necatrix* mycosphere in BS. In comparisons with CS, the fungal OTUs affiliated with *Phialophora*, *Clonostachys*, *Fusarium*, *Geminibasidium*, *Mortierellaceae*, *Nectriaceae*, and *Didymellaceae* were more abundant in the pathogen mycosphere of BS. Many species belonging to *Phialophora*, *Fusarium*, *Nectriaceae*, and *Didymellaceae* are opportunistic or phytopathogens (Kondo *et al.*, 2007; Aveskamp *et al.*, 2010; Aoki *et al.*, 2014), whereas those associated with *Clonostachys* and *Mortierellaceae* have suppressive potential against plant diseases (Sun *et al.*, 2020; Wang *et al.*, 2022). However, the specific roles of these fungal groups warrant further study with respect to the disease development of *R. necatrix*. Among prokaryotes, the percentage of OTUs affiliated with *Dokdonella*, *Variovorax*, *Niastella*, *Geothrix*, and *Rhodanobacter* was enriched in the pathogen mycosphere of BS. The growth of these bacterial groups prefers an optimal pH range of 6.5~7.5 (Yoon *et al.*, 2006a, 2006b; Cho *et al.*, 2017; Akter *et al.*, 2021; Itoh *et al.*, 2023). Due to the higher pH in BS, the change in the *R. necatrix* mycosphere community appears to be linked to the increased soil pH.

In our attempt to link the microbial indicators of the mycosphere community with antagonism against *R. necatrix*, correlation ana­lyses showed that the *R. necatrix* extinction zone in the mycelial toothpick appeared to be associated with fungal and prokaryotic community structures. Our results further suggest that several specific microbial groups present in *R. necatrix* mycosphere increases alongside antagonistic potential. The *R. necatrix* extinction zone significantly increased as the abundance of *Chaetomiaceae* and *Trichoderma* became higher, suggesting that these groups possess antagonistic potential against *R. necatrix*. These results are consistent with previous findings showing the usability of these fungi as biocontrol agents against soil-borne pathogenic fungi, such as *Fusarium* spp., *V. dahliae*, and *R. necatrix* (Carrero-Carrón *et al.*, 2016; Madbouly and Abdel-Wareth, 2020; Takahashi *et al.*, 2020; Kumari *et al.*, 2022). Regarding prokaryotes, the relative abundance of some unclassified bacteria positively correlated with the antagonistic potential, indicating that some unidentified bacteria contribute to the control of *R. necatrix*. However, the percentages of these fungal and bacterial groups with antagonistic potential decreased at different levels in *R. necatrix* mycosphere in BS, which may be attributed to shifts in the soil microenvironment and pathogen vigor. An increasing number of studies have revealed that soil-borne pathogenic fungi release various biomolecules to target the immune system of the host plant and selectively manipulate the local microbiome during host-infecting and soil-dwelling life stages (Snelders *et al.*, 2020; Snelders *et al.*, 2021). To date, dozens of genes encoding antimicrobial effector proteins have been identified in the genome of *R. necatrix*, some of which are expressed to inhibit antagonistic bacteria during host colonization (Chavarro-Carrero *et al.*, 2024). The present results revealed a marked decline in soil antagonism against white root rot disease in BS, which may be partially explained by the selective exclusion of antagonists from the mycosphere community by *R. necatrix*.

In addition, several specific microbial groups enriched in *R. necatrix* mycosphere negatively correlated with the antagonistic potential, which mainly included fungi affiliated with *Phialophora*, *Mortierellaceae*, and *Nectriaceae* and bacteria affiliated with *Dokdonella* and *Rhodanobacteraceae*. This result suggests that low antagonism against *R. necatrix* in BS was also associated with an increased abundance of other co-generic species; however, the specific roles of these microbes in promoting white root rot disease warrant further study. Based on the present results showing differences between BS and CS with respect to antagonism against *R. necatrix* and microbial communities in the pathogen mycosphere, the development of a synthesized approach for the prevention and control of *R. necatrix* that is adapted to the carbon-sinking cultivation system with pruned branch biochar is needed. Future studies are required to focus on enhancing the suppression of *R. necatrix* in BS, particularly by enriching indigenous antagonists or exogenously supplying biocontrol agents.

Previous studies detected and quantified *R. necatrix* in orchard soils using culture-based and mole­cular methods. However, it is typically detectable in infested orchards (Shishido *et al.*, 2012; Arjona-López *et al.*, 2019a; Hartley *et al.*, 2022), particularly close to the trunk (Eguchi *et al.*, 2009). The spread of *R. necatrix* in soil is mainly dependent on the mycelial colonization of healthy roots from adjacent diseased roots through direct root contact between host plants (Pliego *et al.*, 2012). Additionally, *R. necatrix* isolates recovered from diseased roots show higher virulence than those recovered from the soil (Ikeda *et al.*, 2005). These findings imply that the proper management of root-associated soil may be important for the efficient prevention of white root rot in orchards. Although the present study showed that the biochar amendment decreased soil antagonism against *R. necatrix *to some extent, the results obtained also indicate that *R. necatrix* did not grow using pruned branch biochar as a nutrient source (Supplementary Fig. S3). Therefore, the disease may be controlled if biochar is applied to an appropriate location without the root system of the fruit tree. We suggest applying pruned branch biochar to soil away from the tree trunk at a distance far from root-associated soil, which will avoid increasing the risk of white root rot in orchards. In addition, prior to the application of biochar, the detection and quantification of *R. necatrix* in the targeted orchard is required to decide whether to consider the use of biochar.

## Conclusion

We showed that soil antagonism against the white root rot fungus *R. necatrix* decreased in soils amended with 20% (v/v) biochar. Soil pH was neutralized and aeration was promoted by the biochar amendment, which may be favorable for disease occurrence. An investigation of microbial communities surrounding *R. necatrix* mycelia indicated that the antagonistic fungi affiliated with *Chaetomiaceae* and *Trichoderma* were selectively excluded from the mycosphere community in BS. Therefore, we propose that the enrichment of these indigenous antagonistic fungi may be particularly important for controlling white root rot in BS. Further studies enhancing the environmental adaptation of the soil microbiome may open new avenues for the development of information-based biocontrol strategies for *R. necatrix* that are suitable for carbon-sinking cultivation systems. We do not recommend the application of pruned branch biochar to the soil area associated with the roots of fruit trees in order to avoid increasing the risk of white root rot in orchards.

## Citation

Guo, Y., Horii, S., and Kanematsu, S. (2024) Evaluation of Soil Antagonism against the White Root Rot Fungus *Rosellinia necatrix* and Pathogen Mycosphere Communities in Biochar-amended Soil. *Microbes Environ ***39**: ME24060.

https://doi.org/10.1264/jsme2.ME24060

## Supplementary Material

Supplementary Material

## Figures and Tables

**Table 1.** Antagonism against* Rosellinia necatrix* (*Rn*) in orchard soils amended with 20% of pruned branch biochar.

Soil sample, Orchard name, vegetation type, sampling time	*Rn* strain	Length of extinction zone (mm)^a^		
		Biochar-amended	Control	*P* value^b^
S1, Tsukuba-A, low grass cover, May 2023	W97	10.0±2.8	14.5±3.0	0.133
	W563	3.1±1.8	9.0±2.8	0.046 *
S2, Tsukuba-A, low grass cover, July 2023	W97	1.1±0.5	4.6±0.7	0.004 **
	W563	0.3±0.1	1.4±0.2	0.009 **
S3, Tsukuba-B1, low grass cover, October 2023	W97	0.8±0.7	2.3±0.9	0.111
	W563	0.0±0.1	0.6±0.1	0.061
S4, Tsukuba-B2, high grass cover, October 2023	W97	0.1±0.1	3.5±1.5	0.037 *
	W563	0.1±0.1	2.5±0.4	0.001 **
S5, Tsukuba-C, no grass cover, October 2023	W97	3.7±2.4	9.9±2.4	0.008 **
	W563	2.3±0.7	6.5±2.3	0.075
S6, Chiba, no grass cover, August 2023	W97	0.3±0.2	2.8±0.7	0.021 *
	W563	0.3±0.3	1.4±0.2	0.039 *

^a^ Data for the length of the extinction zone in *R. necatrix* mycelial toothpicks represent the mean and standard deviation of triplicate measurements. ^b^ Significant differences between the biochar-amended and control groups were exami­ned with a given comparison (the Student’s *t*-test: * *P*<0.05; ** *P*<0.01).

**Table 2.** Disease severity of white root rot in orchard soils amended with 20% of pruned branch biochar.

Experiment	*Rosellinia necatrix* (*Rn*) strain	Disease severity^a^ of white root rot in the tested plant						
		Mung seedling^b^				Apple plant^c^		
		Biochar-amended	Control	*P* value^d^		Biochar-amended	Control	*P* value^d^
Repeat 1	W97	1.9±0.4	0.2±0.1	<0.001 ***		3.0±0.0	0.8±0.5	0.028 *
	W563	3.0±0.0	1.9±0.3	<0.001 ***		2.8±0.2	2.5±0.3	1.000
Repeat 2	W97	1.9±0.3	0.3±0.1	<0.001 ***		2.7±0.2	2.0±0.5	1.000
	W563	0.7±0.1	0.1±0.1	0.001 **		3.0±0.0	2.8±0.2	1.000

^a^ Disease severity is estimated using the disease scores rated as follows: 0, healthy; 1, wilting of <50% leaves; 2, wilting of ≥50% leaves; 3, entirely withered and dead. ^b^ Data for mung represent the mean and standard error (SE) of 14 tested seedlings at 11 days post-inoculation (dpi). ^c^ Data for apple represent the mean and SE of 6 tested plants at 38 dpi. ^d^ Significant differences between the biochar-amended and control groups were exami­ned with a given comparison (Fisher’s exact test: * *P*<0.05; ** *P*<0.01; *** *P*<0.001).

**Table 3.** Physicochemical properties of orchard soils amended with 20% of pruned branch biochar.

Soil sample, orchard name, vegetation type, and sampling time	TC (g kg^–1^)^a^				TN (g kg^–1^)^a^				Water content (%)^b^				pH^c^		
	Biochar-amended	Control	*P* value^d^		Biochar-amended	Control	*P* value^d^		Biochar-amended	Control	*P* value^e^		Biochar-amended	Control	*P* value^d^
S1, Tsukuba-A, low grass cover, May 2023	101.5±6.4	51.4±0.3	0.005**		4.4±0.1	3.9±0.0	0.005**		26.9±0.8	38.1±0.2	0.100		6.60±0.05	5.25±0.05	<0.001***
S2, Tsukuba-A, low grass cover, July 2023	84.4±1.2	48.4±0.3	<0.001***		4.1±0.1	3.7±0.0	0.001**		38.1±0.5	40.6±0.7	0.100		7.58±0.17	5.92±0.01	0.004**
S3, Tsukuba-B1, low grass cover, October 2023	84.5±5.1	34.1±0.2	0.007**		3.5±0.1	2.9±0.0	0.003**		ND	ND	NA		7.00	5.41	NA
S4, Tsukuba-B2, high grass cover, October 2023	76.0±2.6	33.7±1.5	0.003**		3.3±0.0	2.9±0.2	<0.001***		ND	ND	NA		6.95	5.15	NA
S5, Tsukuba-C, no grass cover, October 2023	123.5±6.3	49.4±0.9	<0.001***		4.9±0.0	4.3±0.1	0.035*		ND	ND	NA		7.28	5.42	NA
S6, Chiba, no grass cover, August 2023	76.5±4.9	45.1±0.6	0.002**		4.1±0.1	3.7±0.1	0.006**		33.8±1.2	35.5±0.8	0.100		7.22±0.08	5.42±0.07	<0.001***

^a^ Data for TC and TN represent the mean and standard deviation (SD) of triplicate measurements. ^b^ Data for the water content of samples S1, S2, and S6 represent the mean and SD of triplicate measurements. ND means ‘not determined’. ^c^ Data for the pH of samples S1, S2, and S6 represent the mean and SD of triplicate measurements, and those for samples S3, S4, and S5 represent a single measurement. ^d^ Significant differences between the biochar-amended and control groups were exami­ned with a given comparison (the Student’s *t*-test: * *P*<0.05; ** *P*<0.01; *** *P*<0.001). NA means ‘not available’. ^e^ Significant differences between the biochar-amended and control groups were exami­ned with a given comparison (the Mann-Whitney U test: * *P*<0.05). NA means ‘not available’.

**Table 4.** Fungal and prokaryotic diversities of *Rosellinia necatrix* (*Rn*) mycosphere communities in orchard soils amended with 20% of pruned branch biochar.

Diversity index^a^	*Rn* strain	Fungi				Bacteria		
		Biochar-amended	Control	*P* value^b^		Biochar-amended	Control	*P* value^b^
OTU count	W97	376±10	338±36	0.322		4328±271	4192±194	0.521
	W563	355±19	341±1	0.207		4071±616	4250±456	0.708
	*Rn*-free	1013	1005	N.A.		4195	4638	N.A.
Chao1 richness	W97	1177±119	1249±72	0.589		6294±390	6094±364	0.552
	W563	1166±4	1247±220	0.435		6158±1332	6310±1094	0.886
	*Rn*-free	1973	1794	N.A.		6113	7140	N.A.
Shannon–Wiener index	W97	2.28±0.20	1.94±0.18	0.001**		6.01±0.19	5.93±0.10	0.539
	W563	2.16±0.06	1.71±0.07	0.088		5.46±0.87	5.90±0.07	0.477
	*Rn*-free	3.58	3.72	N.A.		6.17	6.02	N.A.

^a^ Diversity data for *R. necatrix* mycosphere communities represent the mean and standard deviation of triplicate measurements, and those for *Rn*-free samples represent a single measurement. ^b^ Significant differences between the biochar-amended and control groups were exami­ned with a given comparison (the Student’s *t*-test: * *P*<0.05; ** *P*<0.01). N.A. means ‘not available’.

**Table 5.** Linear and monotonic relationships^a^ between microbial indicators of the *Rosellinia necatrix* mycosphere and the length of the extinction zone.

*R. necatrix* mycosphere microbial indicators	Linear correlations			Monotonic correlations	
	R value	*P* value^b^		R value	*P* value^b^
Fungal OTU count	–0.48	0.117		–0.35	0.272
Fungal Shannon-Wiener index	–0.66	0.020*		–0.66	0.018*
Fungal Chao1 richness	0.22	0.491		0.12	0.721
Fungal structure (PCoA1)	0.83	0.001**		0.78	0.003**
Fungal structure (PCoA2)	–0.63	0.028*		–0.62	0.033*
Prokaryotic OTU count	0.05	0.883		0.09	0.778
Prokaryotic Shannon-Wiener index	0.29	0.364		–0.08	0.795
Prokaryotic Chao1 richness	–0.06	0.857		0.20	0.527
Prokaryotic structure (PCoA1)	–0.60	0.038*		–0.46	0.130
Prokaryotic structure (PCoA2)	–0.52	0.084		–0.61	0.036*

^a^ Linear and monotonic relationships were estimated using Pearson’s and Spearman’s correlations, respectively. ^b^ Correlations were exami­ned using Holm’s method at * *P*<0.05 and ** *P*<0.01.
