# Supplementary material for: Evaluation of Soil Antagonism against the White Root Rot Fungus Rosellinia necatrix and Pathogen Mycosphere Communities in Biochar-amended Soil
Source: Microbes Environ. 2024 Dec 20;39(4):ME24060. doi: 10.1264/jsme2.ME24060 (PMC11821764; doi:10.1264/jsme2.ME24060)
Supplement: Supplementary file 1 — Supplementary Material [file 39_24060_s1.pdf]

Table S1 linear discriminant analysis effect size (LEfSe) calculated the LDA scores for differentially abundant fungal and prokaryotic OTUs in the mycosphere community of *R. necatrix* between soils with and without biochar.

Organism	OTU ID	logMaxMean	Class	LDA	pValue	Taxonomy
	Otu0001	5.720	Rn-M in CS	5.0	0.008	k__Fungi(100);p__Ascomycota(100);c__Sordariomycetes(100);o__Sordariales(100);f__Chaetomiaceae(100);f__Chaetomiaceae_unclassified(93);f__Chaetomiaceae_unclassified(93);
	Otu0002	5.321	Rn-M in BS	4.7	0.016	k__Fungi(100);p__Ascomycota(100);c__Sordariomycetes(100);o__Hypocreales(100);f__Bionectriaceae(100);g__Clonostachys(89);g__Clonostachys_unclassified(86);
	Otu0004	3.886	Rn-M in BS	3.3	0.037	k__Fungi(100);p__Basidiomycota(100);c__Geminibasidiomycetes(100);o__Geminibasidiales(100);f__Geminibasidiaceae(100);g__Geminibasidium(100);s__Geminibasidium_sp(96);
	Otu0005	4.990	Rn-M in BS	4.7	0.006	k__Fungi(100);p__Ascomycota(100);c__Eurotiomycetes(99);o__Chaetothyriales(99);f__Herpotrichiellaceae(99);g__Phialophora(99);s__Phialophora_cyclaminis(99);
	Otu0006	4.606	Rn-M in BS	4.0	0.037	k__Fungi(100);p__Mortierellomycota(100);c__Mortierellomycetes(100);o__Mortierellales(100);f__Mortierellaceae(100);f__Mortierellaceae_unclassified(99);f__Mortierellaceae_unclassified(99);
	Otu0009	4.374	Rn-M in BS	3.8	0.025	k__Fungi(100);p__Ascomycota(100);c__Sordariomycetes(100);o__Hypocreales(100);f__Nectriaceae(100);f__Nectriaceae_unclassified(79);f__Nectriaceae_unclassified(79);
	Otu0010	4.453	Rn-M in CS	3.8	0.037	k__Fungi(100);p__Ascomycota(100);c__Sordariomycetes(100);o__Hypocreales(100);f__Hypocreaceae(100);g__Trichoderma(100);g__Trichoderma_unclassified(100);
	Otu0011	4.466	Rn-M in BS	4.0	0.010	k__Fungi(100);p__Ascomycota(100);c__Sordariomycetes(100);o__Sordariales(91);o__Sordariales_unclassified(91);o__Sordariales_unclassified(91);o__Sordariales_unclassified(91);
	Otu0012	4.345	Rn-M in CS	3.9	0.037	k__Fungi(100);p__Ascomycota(100);c__Sordariomycetes(100);o__Chaetosphaeriales(100);f__Chaetosphaeriaceae(100);g__Dictyochaeta(99);s__Dictyochaeta_lithocarpi(99);
	Otu0013	3.488	Rn-M in BS	3.1	0.013	k__Fungi(100);p__Ascomycota(100);c__Dothideomycetes(100);o__Pleosporales(100);f__Didymellaceae(100);f__Didymellaceae_unclassified(100);f__Didymellaceae_unclassified(100);
	Otu0014	3.951	Rn-M in BS	3.5	0.004	k__Fungi(100);p__Ascomycota(100);c__Sordariomycetes(100);o__Hypocreales(100);f__Nectriaceae(100);g__Fusarium(100);g__Fusarium_unclassified(95);
	Otu0019	2.867	Rn-M in BS	2.4	0.020	k__Fungi(100);p__Ascomycota(100);c__Dothideomycetes(100);o__Pleosporales(100);f__Pleosporaceae(100);g__Alternaria(100);s__Alternaria_prunicola(94);
	Otu0020	3.855	Rn-M in CS	3.2	0.037	k__Fungi(100);p__Ascomycota(100);c__Sordariomycetes(100);o__Hypocreales(100);f__Hypocreaceae(100);g__Trichoderma(100);g__Trichoderma_unclassified(70);
	Otu0022	3.899	Rn-M in BS	3.5	0.010	k__Fungi(100);k__Fungi_unclassified(100);k__Fungi_unclassified(100);k__Fungi_unclassified(100);k__Fungi_unclassified(100);k__Fungi_unclassified(100);k__Fungi_unclassified(100);
	Otu0025	2.709	Rn-M in BS	2.4	0.002	k__Fungi(100);p__Ascomycota(100);c__Sordariomycetes(100);o__Glomerellales(100);f__Plectosphaerellaceae(100);g__Chordomyces(99);s__Chordomyces_antarcticus(99);
Fungi	Otu0026	2.596	Rn-M in BS	2.0	0.015	k__Fungi(100);p__Basidiomycota(100);c__Geminibasidiomycetes(100);o__Geminibasidiales(100);f__Geminibasidiaceae(100);g__Geminibasidium(100);g__Geminibasidium_unclassified(54);
	Otu0030	3.215	Rn-M in BS	2.8	0.030	k__Fungi(100);p__Ascomycota(100);c__Dothideomycetes(100);o__Capnodiales(100);f__Cladosporiaceae(100);g__Cladosporium(100);s__Cladosporium_tenuissimum(83);
	Otu0031	3.107	Rn-M in BS	2.5	0.037	k__Fungi(100);p__Ascomycota(100);c__Sordariomycetes(100);o__Hypocreales(100);f__Nectriaceae(100);g__Fusarium(100);g__Fusarium_unclassified(100);
	Otu0033	3.072	Rn-M in BS	2.7	0.004	k__Fungi(100);p__Ascomycota(100);c__Sordariomycetes(100);o__Hypocreales(100);f__Nectriaceae(100);g__Fusarium(100);s__Fusarium_tricinctum(100);
	Otu0035	2.503	Rn-M in BS	2.2	0.003	k__Fungi(100);p__Ascomycota(100);c__Sordariomycetes(100);o__Glomerellales(100);f__Plectosphaerellaceae(100);g__Gibellulopsis(64);s__Gibellulopsis_sp(63);
	Otu0038	3.410	Rn-M in BS	2.9	0.006	k__Fungi(100);p__Ascomycota(100);c__Eurotiomycetes(100);o__Chaetothyriales(100);f__Herpotrichiellaceae(100);g__Exophiala(100);s__Exophiala_equina(94);
	Otu0042	2.880	Rn-M in CS	2.6	0.004	k__Fungi(100);p__Ascomycota(100);c__Dothideomycetes(100);o__Pleosporales(100);o__Pleosporales_unclassified(100);o__Pleosporales_unclassified(100);o__Pleosporales_unclassified(100);
	Otu0046	2.364	Rn-M in BS	2.0	0.018	k__Fungi(100);p__Ascomycota(100);c__Sordariomycetes(100);o__Glomerellales(100);f__Plectosphaerellaceae(100);g__Plectosphaerella(100);g__Plectosphaerella_unclassified(100);
	Otu0052	3.314	Rn-M in CS	2.9	0.007	k__Fungi(100);p__Ascomycota(100);c__Sordariomycetes(100);o__Chaetosphaeriales(100);f__Chaetosphaeriaceae(100);f__Chaetosphaeriaceae_unclassified(99);f__Chaetosphaeriaceae_unclassified(99);
	Otu0056	3.047	Rn-M in BS	2.6	0.016	k__Fungi(100);p__Mortierellomycota(100);c__Mortierellomycetes(100);o__Mortierellales(100);f__Mortierellaceae(100);f__Mortierellaceae_unclassified(100);f__Mortierellaceae_unclassified(100);
	Otu0066	2.624	Rn-M in BS	2.3	0.006	k__Fungi(100);p__Ascomycota(100);c__Sordariomycetes(100);o__Hypocreales(100);f__Nectriaceae(100);g__Fusarium(100);g__Fusarium_unclassified(100);
	Otu0074	2.772	Rn-M in BS	2.5	0.004	k__Fungi(100);p__Ascomycota(100);c__Sordariomycetes(100);o__Sordariales(100);f__Lasiosphaeriaceae(100);g__Cercophora(100);s__Cercophora_coronata(100);
	Otu0087	2.538	Rn-M in BS	2.1	0.005	k__Fungi(100);p__Mortierellomycota(100);c__Mortierellomycetes(100);o__Mortierellales(100);f__Mortierellaceae(100);f__Mortierellaceae_unclassified(100);f__Mortierellaceae_unclassified(100);
	Otu0099	2.411	Rn-M in BS	2.1	0.030	k__Fungi(100);p__Ascomycota(100);c__Sordariomycetes(100);o__Microascales(100);f__Microascaceae(100);g__Pseudallescheria(79);s__Pseudallescheria_boydii(79);
	Otu0103	2.465	Rn-M in CS	2.1	0.029	k__Fungi(100);p__Ascomycota(100);c__Sordariomycetes(100);o__Atractosporales(100);f__Conlariaceae(100);g__Conlarium(100);s__Conlarium_sacchari(100);
	Otu0125	2.508	Rn-M in BS	2.2	0.002	k__Fungi(100);p__Ascomycota(100);p__Ascomycota_unclassified(80);p__Ascomycota_unclassified(80);p__Ascomycota_unclassified(80);p__Ascomycota_unclassified(80);p__Ascomycota_unclassified(80);
	Otu00002	4.659	Rn-M in CS	4.0	0.010	Bacteria(100);Proteobacteria(100);Betaproteobacteria(100);Burkholderiales(100);Burkholderiaceae(100);Paraburkholderia(77);
	Otu00023	3.987	Rn-M in CS	3.5	0.037	Archaea(100);Thaumarchaeota(100);Nitrososphaerales(100);Nitrososphaeraceae(100);Nitrososphaera(100);Nitrososphaera_unclassified(100);
	Otu00018	4.103	Rn-M in CS	3.4	0.025	Bacteria(100);Proteobacteria(100);Betaproteobacteria(100);Burkholderiales(100);Oxalobacteraceae(100);Pseudoduganella(89);
	Otu00041	3.825	Rn-M in BS	3.3	0.004	Bacteria(100);Proteobacteria(100);Gammaproteobacteria(100);Xanthomonadales(100);Rhodanobacteraceae(100);Dokdonella(100);
	Otu00024	3.945	Rn-M in CS	3.3	0.016	Bacteria(100);Bacteria_unclassified(100);Bacteria_unclassified(100);Bacteria_unclassified(100);Bacteria_unclassified(100);Bacteria_unclassified(100);
	Otu00035	3.823	Rn-M in CS	3.2	0.004	Bacteria(100);Bacteria_unclassified(100);Bacteria_unclassified(100);Bacteria_unclassified(100);Bacteria_unclassified(100);Bacteria_unclassified(100);
	Otu00021	3.944	Rn-M in CS	3.2	0.037	Bacteria(100);Acidobacteria(100);Acidobacteria_Gp2(100);Gp2(100);Gp2_unclassified(100);Gp2_unclassified(100);
	Otu00047	3.731	Rn-M in CS	3.2	0.004	Bacteria(100);Acidobacteria(100);Acidobacteria_Gp2(100);Gp2(100);Gp2_unclassified(100);Gp2_unclassified(100);
	Otu00033	3.886	Rn-M in BS	3.1	0.037	Bacteria(100);Proteobacteria(100);Betaproteobacteria(100);Burkholderiales(100);Comamonadaceae(100);Variovorax(75);
	Otu00043	3.791	Rn-M in CS	3.1	0.004	Bacteria(100);Proteobacteria(100);Alphaproteobacteria(100);Alphaproteobacteria_unclassified(80);Alphaproteobacteria_unclassified(80);Alphaproteobacteria_unclassified(80);
	Otu00037	3.731	Rn-M in CS	3.1	0.037	Archaea(100);Thaumarchaeota(100);Thaumarchaeota_unclassified(100);Thaumarchaeota_unclassified(100);Thaumarchaeota_unclassified(100);Thaumarchaeota_unclassified(100);
	Otu00031	3.805	Rn-M in CS	3.1	0.037	Bacteria(100);Acidobacteria(100);Acidobacteria_Gp2(100);Gp2(100);Gp2_unclassified(100);Gp2_unclassified(100);
	Otu00056	3.651	Rn-M in CS	3.1	0.037	Archaea(100);Thaumarchaeota(100);Nitrososphaerales(100);Nitrososphaeraceae(100);Nitrososphaera(100);Nitrososphaera_unclassified(100);
	Otu00136	3.389	Rn-M in CS	3.0	0.004	Bacteria(100);Proteobacteria(100);Deltaproteobacteria(100);Myxococcales(100);Polyangiaceae(100);Polyangiaceae_unclassified(100);
	Otu00116	3.396	Rn-M in BS	3.0	0.004	Bacteria(100);Bacteroidetes(100);Chitinophagia(100);Chitinophagales(100);Chitinophagaceae(100);Niastella(100);
	Otu00082	3.482	Rn-M in BS	3.0	0.004	Bacteria(100);Acidobacteria(100);Holophagae(100);Holophagales(100);Holophagaceae(100);Geothrix(100);
	Otu00122	3.303	Rn-M in BS	3.0	0.004	Bacteria(100);Proteobacteria(100);Gammaproteobacteria(100);Xanthomonadales(100);Rhodanobacteraceae(100);Rhodanobacter(100);
	Otu00059	3.618	Rn-M in CS	3.0	0.010	Bacteria(100);Acidobacteria(100);Acidobacteria_Gp2(100);Gp2(100);Gp2_unclassified(100);Gp2_unclassified(100);
	Otu00053	3.608	Rn-M in BS	2.9	0.006	Bacteria(100);Proteobacteria(100);Betaproteobacteria(100);Betaproteobacteria_unclassified(100);Betaproteobacteria_unclassified(100);Betaproteobacteria_unclassified(100);
	Otu00054	3.606	Rn-M in CS	2.9	0.037	Bacteria(100);Acidobacteria(100);Acidobacteria_Gp2(100);Gp2(100);Gp2_unclassified(100);Gp2_unclassified(100);
	Otu00186	3.190	Rn-M in BS	2.9	0.004	Bacteria(100);Proteobacteria(100);Deltaproteobacteria(100);Myxococcales(100);Myxococcales_unclassified(96);Myxococcales_unclassified(96);

	Otu00139	3.283	Rn-M in CS	2.8	0.004 Bacteria(100);Actinobacteria(100);Actinobacteria(100);Catenulisporales(100);Catenulisporaceae(100);Catenulispora(100);
	Otu00045	3.649	Rn-M in CS	2.8	0.025 Bacteria(100);Bacteria_unclassified(100);Bacteria_unclassified(100);Bacteria_unclassified(100);Bacteria_unclassified(100);Bacteria_unclassified(100);
	Otu00141	3.197	Rn-M in BS	2.8	0.004 Bacteria(100);Bacteroidetes(100);Chitinophagia(100);Chitinophagales(100);Chitinophagaceae(100);Chitinophagaceae_unclassified(100);
	Otu00063	3.544	Rn-M in CS	2.8	0.025 Bacteria(100);Proteobacteria(100);Betaproteobacteria(100);Betaproteobacteria_unclassified(100);Betaproteobacteria_unclassified(100);Betaproteobacteria_unclassified(100);
	Otu00125	3.246	Rn-M in BS	2.8	0.016 Bacteria(100);Actinobacteria(100);Actinobacteria(100);Micrococcales(100);Micrococcaceae(100);Pseudarthrobacter(100);
	Otu00067	3.544	Rn-M in CS	2.8	0.004 Bacteria(100);Proteobacteria(100);Alphaproteobacteria(100);Micropepsales(99);Micropepsaceae(99);Micropepsaceae_unclassified(99);
	Otu00103	3.359	Rn-M in BS	2.7	0.025 Bacteria(100);Actinobacteria(100);Actinobacteria(100);Micrococcales(100);Micrococcaceae(100);Arthrobacter(92);
	Otu00092	3.370	Rn-M in BS	2.7	0.006 Bacteria(100);Proteobacteria(100);Gammaproteobacteria(100);Xanthomonadales(100);Rhodanobacteraceae(100);Rhodanobacteraceae_unclassified(53);
	Otu00187	3.127	Rn-M in CS	2.7	0.025 Archaea(100);Euryarchaeota(100);Thermoplasmata(100);Thermoplasmata_unclassified(84);Thermoplasmata_unclassified(84);Thermoplasmata_unclassified(84);
	Otu00166	3.149	Rn-M in BS	2.7	0.006 Bacteria(100);Bacteroidetes(100);Chitinophagia(100);Chitinophagales(100);Chitinophagaceae(100);Niastella(100);
	Otu00138	3.213	Rn-M in BS	2.7	0.037 Bacteria(100);Bacteroidetes(100);Chitinophagia(100);Chitinophagales(100);Chitinophagaceae(100);Chitinophaga(100);
	Otu00245	3.054	Rn-M in CS	2.6	0.004 Bacteria(100);Proteobacteria(100);Deltaproteobacteria(100);Myxococcales(100);Polyangiaceae(100);Polyangiaceae_unclassified(98);
	Otu00140	3.230	Rn-M in CS	2.6	0.010 Bacteria(100);Proteobacteria(100);Betaproteobacteria(100);Burkholderiales(100);Oxalobacteraceae(100);Massilia(100);
	Otu00084	3.424	Rn-M in BS	2.6	0.010 Bacteria(100);Proteobacteria(100);Alphaproteobacteria(100);Rhodospirillales(100);Reyranellaceae(100);Reyranella(100);
	Otu00137	3.120	Rn-M in BS	2.6	0.004 Bacteria(100);Acidobacteria(100);Acidobacteria_Gp4(100);Gp4(100);Gp4_unclassified(100);Gp4_unclassified(100);
	Otu00189	3.133	Rn-M in CS	2.6	0.004 Bacteria(100);Proteobacteria(100);Gammaproteobacteria(100);Gammaproteobacteria_incertae_sedis(100);Acidibacter(100);Acidibacter_unclassified(100);
	Otu00183	3.119	Rn-M in BS	2.6	0.004 Bacteria(100);Bacteroidetes(100);Chitinophagia(100);Chitinophagales(100);Chitinophagaceae(100);Chitinophagaceae_unclassified(100);
	Otu00110	3.301	Rn-M in CS	2.6	0.045 Bacteria(100);Bacteria_unclassified(100);Bacteria_unclassified(100);Bacteria_unclassified(100);Bacteria_unclassified(100);Bacteria_unclassified(100);
	Otu00340	2.941	Rn-M in BS	2.6	0.004 Bacteria(100);Proteobacteria(100);Gammaproteobacteria(100);Xanthomonadales(100);Xanthomonadaceae(100);Lysobacter(55);
	Otu00343	2.901	Rn-M in BS	2.6	0.004 Bacteria(100);Proteobacteria(100);Gammaproteobacteria(100);Xanthomonadales(100);Xanthomonadaceae(100);Stenotrophomonas(100);
	Otu00090	3.321	Rn-M in CS	2.6	0.037 Bacteria(100);Acidobacteria(100);Acidobacteria_Gp2(100);Gp2(100);Gp2_unclassified(100);Gp2_unclassified(100);
	Otu00121	3.252	Rn-M in CS	2.5	0.016 Bacteria(100);Proteobacteria(100);Alphaproteobacteria(100);Caulobacterales(100);Caulobacteraceae(100);Caulobacteraceae_unclassified(97);
	Otu00226	2.933	Rn-M in BS	2.5	0.004 Bacteria(100);Gemmatimonadetes(100);Gemmatimonadetes(100);Gemmatimonadales(100);Gemmatimonadaceae(100);Gemmatimonas(70);
	Otu00129	3.145	Rn-M in BS	2.5	0.004 Bacteria(100);Acidobacteria(100);Acidobacteria_Gp16(100);Gp16(100);Gp16_unclassified(100);Gp16_unclassified(100);
	Otu00173	3.039	Rn-M in BS	2.5	0.010 Bacteria(100);Acidobacteria(100);Acidobacteria_Gp6(100);Gp6(100);Gp6_unclassified(100);Gp6_unclassified(100);
	Otu00162	3.086	Rn-M in BS	2.5	0.004 Bacteria(100);Proteobacteria(100);Alphaproteobacteria(100);Rhizobiales(100);Hyphomicrobiaceae(100);Pedomicrobium(76);
	Otu00222	2.993	Rn-M in BS	2.5	0.025 Bacteria(100);Actinobacteria(100);Actinobacteria(100);Micromonosporales(98);Micromonosporaceae(98);Dactylosporangium(77);
	Otu00337	2.874	Rn-M in CS	2.5	0.004 Bacteria(100);Verrucomicrobia(100);Subdivision3(100);Subdivision3_unclassified(100);Subdivision3_unclassified(100);Subdivision3_unclassified(100);
	Otu00270	2.865	Rn-M in BS	2.4	0.004 Bacteria(100);Acidobacteria(100);Blastocatellia(100);Blastocatellales(100);Blastocatellaceae(100);Blastocatellaceae_unclassified(99);
	Otu00205	2.994	Rn-M in CS	2.4	0.006 Bacteria(100);Acidobacteria(100);Acidobacteria_Gp2(100);Gp2(100);Gp2_unclassified(100);Gp2_unclassified(100);
	Otu00230	3.019	Rn-M in CS	2.4	0.013 Bacteria(100);Proteobacteria(100);Alphaproteobacteria(100);Sphingomonadales(100);Sphingomonadaceae(100);Sphingobium(98);
	Otu00224	2.925	Rn-M in BS	2.4	0.006 Bacteria(100);Actinobacteria(100);Actinobacteria(100);Micromonosporales(100);Micromonosporaceae(100);Micromonosporaceae_unclassified(95);
	Otu00124	3.025	Rn-M in BS	2.4	0.030 Bacteria(100);Acidobacteria(100);Acidobacteria_Gp4(100);Gp4(100);Gp4_unclassified(100);Gp4_unclassified(100);
	Otu00184	3.048	Rn-M in CS	2.4	0.030 Bacteria(100);Acidobacteria(100);Acidobacteria_Gp1(100);Gp1(100);Gp1_unclassified(100);Gp1_unclassified(100);
	Otu00477	2.692	Rn-M in BS	2.4	0.003 Bacteria(100);Actinobacteria(100);Actinobacteria(100);Streptosporangiales(100);Streptosporangiaceae(100);Acrocarpospora(66);
	Otu00450	2.676	Rn-M in BS	2.4	0.003 Bacteria(100);Bacteria_unclassified(100);Bacteria_unclassified(100);Bacteria_unclassified(100);Bacteria_unclassified(100);Bacteria_unclassified(100);
	Otu00511	2.671	Rn-M in BS	2.4	0.003 Bacteria(100);Actinobacteria(100);Actinobacteria(100);Streptosporangiales(100);Streptosporangiaceae(100);Nonomuraea(100);
	Otu00113	3.242	Rn-M in CS	2.4	0.020 Bacteria(100);Proteobacteria(100);Alphaproteobacteria(100);Rhodospirillales(100);Rhodospirillales_unclassified(100);Rhodospirillales_unclassified(100);
Prokaryotes	Otu00154	3.080	Rn-M in BS	2.4	0.006 Bacteria(100);Proteobacteria(100);Gammaproteobacteria(100);Xanthomonadales(100);Xanthomonadales_unclassified(100);Xanthomonadales_unclassified(100);
	Otu00180	2.934	Rn-M in CS	2.4	0.020 Bacteria(100);Acidobacteria(100);Acidobacteria_Gp2(100);Gp2(100);Gp2_unclassified(100);Gp2_unclassified(100);
	Otu00242	2.838	Rn-M in BS	2.4	0.010 Bacteria(100);Verrucomicrobia(100);Opitutae(100);Opitutales(100);Opitutaceae(100);Lacunisphaera(100);
	Otu00388	2.747	Rn-M in BS	2.4	0.004 Bacteria(100);Bacteroidetes(100);Chitinophagia(100);Chitinophagales(100);Chitinophagaceae(100);Chitinophagaceae_unclassified(100);
	Otu00417	2.676	Rn-M in BS	2.4	0.004 Bacteria(100);Actinobacteria(100);Actinobacteria(100);Propionibacteriales(100);Nocardioidaceae(100);Nocardioides(100);
	Otu00346	2.768	Rn-M in BS	2.3	0.004 Bacteria(100);Proteobacteria(100);Gammaproteobacteria(100);Nevskiales(100);Steroidobacteraceae(100);Povalibacter(87);
	Otu00272	2.883	Rn-M in BS	2.3	0.013 Bacteria(100);Bacteroidetes(100);Chitinophagia(100);Chitinophagales(100);Chitinophagaceae(100);Chitinophagaceae_unclassified(100);
	Otu00232	2.927	Rn-M in CS	2.3	0.010 Bacteria(100);Proteobacteria(100);Deltaproteobacteria(100);Myxococcales(100);Myxococcales_unclassified(100);Myxococcales_unclassified(100);
	Otu00133	3.067	Rn-M in BS	2.3	0.010 Bacteria(100);Acidobacteria(100);Acidobacteria_Gp16(100);Gp16(100);Gp16_unclassified(100);Gp16_unclassified(100);
	Otu00503	2.689	Rn-M in CS	2.3	0.004 Bacteria(100);Proteobacteria(100);Alphaproteobacteria(100);Alphaproteobacteria_unclassified(100);Alphaproteobacteria_unclassified(100);Alphaproteobacteria_unclassified(100);
	Otu00438	2.675	Rn-M in BS	2.3	0.004 Bacteria(100);Actinobacteria(100);Actinobacteria(100);Propionibacteriales(100);Nocardioidaceae(100);Marmoricola(98);
	Otu00420	2.736	Rn-M in BS	2.3	0.004 Bacteria(100);Bacteroidetes(100);Sphingobacteriia(100);Sphingobacteriales(100);Sphingobacteriaceae(100);Mucilaginibacter(100);
	Otu00175	2.920	Rn-M in BS	2.3	0.016 Bacteria(100);Proteobacteria(100);Alphaproteobacteria(100);Rhodospirillales(100);Rhodospirillales_unclassified(100);Rhodospirillales_unclassified(100);
	Otu00518	2.625	Rn-M in BS	2.3	0.002 Bacteria(100);Firmicutes(100);Bacilli(100);Bacillales(100);Bacillaceae_1(100);Alkalihalobacillus(97);
	Otu00164	3.004	Rn-M in CS	2.3	0.024 Bacteria(100);Bacteria_unclassified(100);Bacteria_unclassified(100);Bacteria_unclassified(100);Bacteria_unclassified(100);Bacteria_unclassified(100);
	Otu00294	2.843	Rn-M in CS	2.3	0.006 Bacteria(100);Proteobacteria(100);Alphaproteobacteria(100);Micropepsales(100);Micropepsaceae(100);Micropepsaceae_unclassified(99);

Otu00591	2.595	Rn-M in CS	2.3	0.004	Bacteria(100);Bacteria_unclassified(100);Bacteria_unclassified(100);Bacteria_unclassified(100);Bacteria_unclassified(100);Bacteria_unclassified(100);
Otu00316	2.798	Rn-M in BS	2.3	0.037	Bacteria(100);Actinobacteria(100);Actinobacteria(100);Streptomycetales(100);Streptomycetaceae(100);Streptacidiphilus(69);
Otu00545	2.588	Rn-M in BS	2.3	0.004	Bacteria(100);Bacteroidetes(100);Sphingobacteriia(100);Sphingobacteriales(100);Sphingobacteriaceae(100);Sphingobacteriaceae_unclassified(99);
Otu00578	2.544	Rn-M in BS	2.3	0.003	Bacteria(100);Gemmatimonadetes(100);Gemmatimonadetes(100);Gemmatimonadales(100);Gemmatimonadaceae(100);Gemmatimonas(100);
Otu00567	2.535	Rn-M in BS	2.3	0.013	Bacteria(100);Bacteroidetes(100);Chitinophagia(100);Chitinophagales(100);Chitinophagaceae(100);Chitinophaga(100);
Otu00118	3.115	Rn-M in CS	2.2	0.037	Bacteria(100);Bacteria_unclassified(100);Bacteria_unclassified(100);Bacteria_unclassified(100);Bacteria_unclassified(100);Bacteria_unclassified(100);
Otu00284	2.846	Rn-M in BS	2.2	0.025	Bacteria(100);Bacteroidetes(100);Chitinophagia(100);Chitinophagales(100);Chitinophagaceae(100);Niastella(100);
Otu00216	2.946	Rn-M in CS	2.2	0.025	Bacteria(100);Proteobacteria(100);Gammaproteobacteria(100);Gammaproteobacteria_incertae_sedis(100);Acidibacter(100);Acidibacter_unclassified(100);
Otu00345	2.795	Rn-M in CS	2.2	0.010	Bacteria(100);Actinobacteria(100);Actinobacteria(100);Streptosporangiales(99);Streptosporangiales_unclassified(99);Streptosporangiales_unclassified(99);
Otu00400	2.726	Rn-M in CS	2.2	0.004	Bacteria(100);Proteobacteria(100);Deltaproteobacteria(74);Deltaproteobacteria_unclassified(74);Deltaproteobacteria_unclassified(74);Deltaproteobacteria_unclassified(74);
Otu00233	2.916	Rn-M in BS	2.2	0.010	Bacteria(100);Proteobacteria(100);Alphaproteobacteria(100);Sphingomonadales(100);Sphingomonadaceae(100);Sphingomonas(100);
Otu00569	2.565	Rn-M in BS	2.2	0.003	Bacteria(100);Proteobacteria(100);Gammaproteobacteria(100);Xanthomonadales(100);Xanthomonadaceae(100);Lysobacter(100);
Otu00177	2.868	Rn-M in BS	2.2	0.025	Bacteria(100);Bacteria_unclassified(100);Bacteria_unclassified(100);Bacteria_unclassified(100);Bacteria_unclassified(100);Bacteria_unclassified(100);
Otu00524	2.616	Rn-M in CS	2.2	0.013	Bacteria(100);Verrucomicrobia(100);Subdivision3(100);Subdivision3_unclassified(100);Subdivision3_unclassified(100);Subdivision3_unclassified(100);
Otu00351	2.758	Rn-M in BS	2.2	0.010	Bacteria(100);Bacteroidetes(100);Chitinophagia(100);Chitinophagales(100);Chitinophagaceae(100);Chitinophagaceae_unclassified(100);
Otu00469	2.660	Rn-M in CS	2.2	0.016	Bacteria(100);Verrucomicrobia(100);Verrucomicrobia_unclassified(100);Verrucomicrobia_unclassified(100);Verrucomicrobia_unclassified(100);Verrucomicrobia_unclassified(100);
Otu00314	2.760	Rn-M in BS	2.2	0.004	Bacteria(100);Actinobacteria(100);Actinobacteria(100);Actinobacteria_unclassified(100);Actinobacteria_unclassified(100);Actinobacteria_unclassified(100);
Otu00282	2.720	Rn-M in BS	2.2	0.006	Bacteria(100);Acidobacteria(100);Acidobacteria_Gp4(100);Gp4(100);Gp4_unclassified(100);Gp4_unclassified(100);
Otu00290	2.820	Rn-M in BS	2.2	0.020	Bacteria(100);Proteobacteria(100);Betaproteobacteria(100);Burkholderiales(100);Comamonadaceae(100);Comamonadaceae_unclassified(69);
Otu00280	2.831	Rn-M in BS	2.2	0.016	Bacteria(100);Proteobacteria(100);Alphaproteobacteria(100);Rhodospirillales(100);Reyranellaceae(100);Reyranella(100);
Otu00380	2.665	Rn-M in BS	2.2	0.004	Bacteria(100);Proteobacteria(100);Betaproteobacteria(100);Nitrosomonadales(98);Thiobacillaceae(98);Thiobacillaceae_unclassified(98);
Otu00281	2.857	Rn-M in CS	2.1	0.006	Bacteria(100);Proteobacteria(100);Betaproteobacteria(100);Nitrosomonadales(99);Nitrosomonadales_unclassified(99);Nitrosomonadales_unclassified(99);
Otu00532	2.580	Rn-M in BS	2.1	0.004	Bacteria(100);Proteobacteria(100);Alphaproteobacteria(100);Rhizobiales(100);Phyllobacteriaceae(100);Phyllobacterium(100);
Otu00506	2.597	Rn-M in CS	2.1	0.025	Bacteria(100);Bacteria_unclassified(100);Bacteria_unclassified(100);Bacteria_unclassified(100);Bacteria_unclassified(100);Bacteria_unclassified(100);
Otu00687	2.506	Rn-M in CS	2.1	0.004	Bacteria(100);Proteobacteria(100);Betaproteobacteria(100);Burkholderiales(100);Oxalobacteraceae(100);Herbaspirillum(100);
Otu00347	2.729	Rn-M in CS	2.1	0.036	Bacteria(100);Proteobacteria(100);Alphaproteobacteria(100);Rhodospirillales(100);Rhodospirillales_unclassified(100);Rhodospirillales_unclassified(100);
Otu00403	2.601	Rn-M in BS	2.1	0.004	Bacteria(100);Proteobacteria(100);Alphaproteobacteria(100);Rhodospirillales(100);Rhodospirillales_unclassified(100);Rhodospirillales_unclassified(100);
Otu00509	2.568	Rn-M in CS	2.1	0.013	Bacteria(100);Proteobacteria(100);Gammaproteobacteria(100);Nevskiales(56);Nevskiales_unclassified(56);Nevskiales_unclassified(56);
Otu00496	2.568	Rn-M in BS	2.1	0.025	Bacteria(100);Actinobacteria(100);Actinobacteria(100);Streptomycetales(100);Streptomycetaceae(100);Yinghuangia(99);
Otu00635	2.455	Rn-M in BS	2.1	0.019	Bacteria(100);Proteobacteria(100);Alphaproteobacteria(100);Caulobacterales(100);Caulobacteraceae(100);Caulobacter(86);
Otu00464	2.636	Rn-M in CS	2.1	0.004	Bacteria(100);Planctomycetes(100);Planctomycetacia(100);Planctomycetales(100);Isosphaeraceae(100);Isosphaeraceae_unclassified(99);
Otu00570	2.466	Rn-M in BS	2.1	0.004	Bacteria(100);Proteobacteria(100);Betaproteobacteria(100);Betaproteobacteria_unclassified(100);Betaproteobacteria_unclassified(100);Betaproteobacteria_unclassified(100);
Otu00743	2.425	Rn-M in CS	2.0	0.004	Bacteria(100);Proteobacteria(100);Deltaproteobacteria(100);Bdellovibrionales(100);Bdellovibrionaceae(100);Bdellovibrio(100);
Otu00613	2.463	Rn-M in BS	2.0	0.004	Bacteria(100);Bacteroidetes(100);Chitinophagia(100);Chitinophagales(100);Chitinophagaceae(100);Ferruginibacter(100);
Otu00558	2.442	Rn-M in BS	2.0	0.022	Bacteria(100);Proteobacteria(100);Betaproteobacteria(100);Betaproteobacteria_unclassified(100);Betaproteobacteria_unclassified(100);Betaproteobacteria_unclassified(100);
Otu00572	2.463	Rn-M in BS	2.0	0.005	Bacteria(100);Actinobacteria(100);Actinobacteria(100);Micromonosporales(100);Micromonosporaceae(100);Micromonosporaceae_unclassified(77);
Otu00706	2.416	Rn-M in BS	2.0	0.004	Bacteria(100);Firmicutes(100);Bacilli(100);Bacillales(100);Bacillales_unclassified(96);Bacillales_unclassified(96);
Otu00357	2.690	Rn-M in CS	2.0	0.006	Bacteria(100);Proteobacteria(100);Alphaproteobacteria(100);Rhodospirillales(100);Rhodospirillales_unclassified(100);Rhodospirillales_unclassified(100);
Otu00361	2.607	Rn-M in CS	2.0	0.016	Bacteria(100);Acidobacteria(100);Acidobacteria_Gp2(100);Gp2(100);Gp2_unclassified(100);Gp2_unclassified(100);
Otu00300	2.710	Rn-M in CS	2.0	0.005	Bacteria(100);Acidobacteria(100);Acidobacteria_Gp2(100);Gp2(100);Gp2_unclassified(100);Gp2_unclassified(100);
Otu00426	2.588	Rn-M in CS	2.0	0.010	Bacteria(100);Bacteria_unclassified(100);Bacteria_unclassified(100);Bacteria_unclassified(100);Bacteria_unclassified(100);Bacteria_unclassified(100);
Otu00593	2.489	Rn-M in CS	2.0	0.004	Bacteria(100);Proteobacteria(100);Deltaproteobacteria(58);Myxococcales(58);Myxococcales_unclassified(58);Myxococcales_unclassified(58);
Otu00262	2.782	Rn-M in BS	2.0	0.016	Bacteria(100);Bacteria_unclassified(100);Bacteria_unclassified(100);Bacteria_unclassified(100);Bacteria_unclassified(100);Bacteria_unclassified(100);
Otu00227	2.822	Rn-M in BS	2.0	0.036	Bacteria(100);Acidobacteria(100);Acidobacteria_Gp4(100);Gp4(100);Gp4_unclassified(100);Gp4_unclassified(100);
Otu00311	2.690	Rn-M in BS	2.0	0.025	Bacteria(100);Acidobacteria(100);Acidobacteria_Gp3(100);Candidatus_Solibacter(71);Candidatus_Solibacter_unclassified(71);Candidatus_Solibacter_unclassified(71);

Table S2 Linear regression relationship (Pearson's correlation) between the specific OTUs and the extinction zone of *R. necatrix* in the mycelial toothpicks.

Organism	OTU ID	p-value	r-value	Relative abundance (%)						Taxonomy
				Rn-free BS	Rn-free CS	W97-M in BS	W97-M in CS	W563-M in BS	W563-M in CS	
Fungi	Otu0001	0.029	0.627	1.59	2.31	36.29	57.30	30.86	47.61	k__Fungi(100);p__Ascomycota(100);c__Sordariomycetes(100);o__Sordariales(100);f__Chaetomiaceae(100);f__Chaetomiaceae_unclassified(93);f__Chaetomiaceae_unclassified(93);
	Otu0006	0.014	-0.687	3.59	3.77	2.31	1.19	5.71	2.00	k__Fungi(100);p__Mortierellomycota(100);c__Mortierellomycetes(100);o__Mortierellales(100);f__Mortierellaceae(100);f__Mortierellaceae_unclassified(99);f__Mortierellaceae_unclassified(99);
	Otu0009	0.001	-0.824	1.62	4.55	1.35	0.85	3.35	0.97	k__Fungi(100);p__Ascomycota(100);c__Sordariomycetes(100);o__Hypocreales(100);f__Nectriaceae(100);f__Nectriaceae_unclassified(79);f__Nectriaceae_unclassified(79);
	Otu0010	0.034	0.612	0.07	0.12	1.77	4.17	0.95	1.48	k__Fungi(100);p__Ascomycota(100);c__Sordariomycetes(100);o__Hypocreales(100);f__Hypocreaceae(100);g__Trichoderma(100);g__Trichoderma_unclassified(100);
	Otu0014	0.012	-0.697	5.08	2.22	0.70	0.30	1.06	0.22	k__Fungi(100);p__Ascomycota(100);c__Sordariomycetes(100);o__Hypocreales(100);f__Nectriaceae(100);g__Fusarium(100);g__Fusarium_unclassified(95);
	Otu0015	0.009	-0.713	0.18	0.34	0.36	0.21	1.66	0.50	k__Fungi(100);k__Fungi_unclassified(71);k__Fungi_unclassified(71);k__Fungi_unclassified(71);k__Fungi_unclassified(71);k__Fungi_unclassified(71);k__Fungi_unclassified(71);
	Otu0019	0.048	-0.581	1.17	4.65	0.03	0.63	0.12	0.02	k__Fungi(100);p__Ascomycota(100);c__Dothideomycetes(100);o__Pleosporales(100);f__Pleosporaceae(100);g__Alternaria(100);s__Alternaria_prunicola(94);
	Otu0022	0.026	-0.636	0.01	0.00	0.48	0.15	1.08	0.14	k__Fungi(100);k__Fungi_unclassified(100);k__Fungi_unclassified(100);k__Fungi_unclassified(100);k__Fungi_unclassified(100);k__Fungi_unclassified(100);k__Fungi_unclassified(100);
	Otu0026	0.003	-0.784	1.82	1.65	0.02	0.01	0.05	0.01	k__Fungi(100);p__Basidiomycota(100);c__Geminibasidiomycetes(100);o__Geminibasidiales(100);f__Geminibasidiaceae(100);g__Geminibasidium(100);g__Geminibasidium_unclassified(54);
	Otu0028	0.012	-0.694	0.96	2.17	0.01	0.02	0.02	0.01	k__Fungi(100);p__Ascomycota(100);c__Sordariomycetes(100);o__Hypocreales(100);f__Nectriaceae(100);g__Thelonectria(100);s__Thelonectria_rubi(90);
	Otu0031	0.004	-0.756	0.80	0.58	0.10	0.01	0.15	0.06	k__Fungi(100);p__Ascomycota(100);c__Sordariomycetes(100);o__Hypocreales(100);f__Nectriaceae(100);g__Fusarium(100);g__Fusarium_unclassified(100);
	Otu0036	0.003	-0.777	0.98	0.76	0.01	0.10	0.04	0.01	k__Fungi(100);p__Mortierellomycota(100);c__Mortierellomycetes(100);o__Mortierellales(100);f__Mortierellaceae(100);g__Mortierella(100);s__Mortierella_pseudozygospora(100);
	Otu0038	0.003	-0.780	0.08	0.05	0.16	0.38	0.35	0.11	k__Fungi(100);p__Ascomycota(100);c__Eurotiomycetes(100);o__Chaetothyriales(100);f__Herpotrichiellaceae(100);g__Exophiala(100);s__Exophiala_equina(94);
	Otu0040	0.040	-0.597	0.30	0.43	0.04	0.04	0.19	0.07	k__Fungi(100);p__Mortierellomycota(100);c__Mortierellomycetes(100);o__Mortierellales(100);f__Mortierellaceae(100);g__Podila(100);s__Podila_minutissima(94);
	Otu0043	0.010	-0.708	0.50	0.88	0.01	0.04	0.02	0.01	k__Fungi(100);p__Ascomycota(100);c__Sordariomycetes(100);o__Hypocreales(100);f__Nectriaceae(100);g__Penicillifer(100);s__Penicillifer_diparietisporus(100);
	Otu0047	0.005	-0.751	0.12	0.95	0.01	0.01	0.03	0.01	k__Fungi(100);p__Ascomycota(100);c__Sordariomycetes(100);o__Xylariales(96);f__Xylariales_fam_Incertae_sedis(91);g__Fusidium(91);s__Fusidium_sp(90);
	Otu0050	0.001	-0.823	0.45	0.49	0.01	0.23	0.01	0.01	k__Fungi(100);p__Ascomycota(100);c__Eurotiomycetes(100);o__Eurotiales(100);f__Aspergillaceae(100);g__Phialomyces(100);s__Phialomyces_macrosporus(100);
Prokaryotes	Otu00004	0.005	0.755	4.34	5.11	3.21	2.95	1.81	2.54	Bacteria(100);Bacteria_unclassified(100);Bacteria_unclassified(100);Bacteria_unclassified(100);Bacteria_unclassified(100);Bacteria_unclassified(100);
	Otu00006	0.028	0.630	0.04	0.23	2.59	3.66	1.79	2.11	Bacteria(100);Proteobacteria(100);Betaproteobacteria(100);Nitrosomonadales(100);Nitrosomonadales_unclassified(100);Nitrosomonadales_unclassified(100);
	Otu00007	0.005	0.747	2.42	2.96	2.19	1.97	1.24	1.76	Bacteria(100);Bacteria_unclassified(100);Bacteria_unclassified(100);Bacteria_unclassified(100);Bacteria_unclassified(100);Bacteria_unclassified(100);
	Otu00010	0.011	0.704	1.87	2.61	1.64	1.43	0.93	1.27	Bacteria(100);Bacteria_unclassified(100);Bacteria_unclassified(100);Bacteria_unclassified(100);Bacteria_unclassified(100);Bacteria_unclassified(100);
	Otu00022	0.036	0.607	0.45	0.37	0.85	0.89	0.40	0.84	Bacteria(100);Bacteria_unclassified(100);Bacteria_unclassified(100);Bacteria_unclassified(100);Bacteria_unclassified(100);Bacteria_unclassified(100);
	Otu00029	0.010	0.705	0.66	0.98	0.67	0.62	0.39	0.57	Bacteria(100);Bacteria_unclassified(100);Bacteria_unclassified(100);Bacteria_unclassified(100);Bacteria_unclassified(100);Bacteria_unclassified(100);
	Otu00037	0.046	0.585	0.97	1.41	0.37	0.55	0.14	0.53	Archaea(100);Thaumarchaeota(100);Thaumarchaeota_unclassified(100);Thaumarchaeota_unclassified(100);Thaumarchaeota_unclassified(100);Thaumarchaeota_unclassified(100);
	Otu00043	0.027	0.632	0.04	0.04	0.36	0.67	0.30	0.57	Bacteria(100);Proteobacteria(100);Alphaproteobacteria(100);Alphaproteobacteria_unclassified(80);Alphaproteobacteria_unclassified(80);Alphaproteobacteria_unclassified(80);
	Otu00045	0.043	0.590	0.38	0.74	0.36	0.49	0.25	0.41	Bacteria(100);Bacteria_unclassified(100);Bacteria_unclassified(100);Bacteria_unclassified(100);Bacteria_unclassified(100);Bacteria_unclassified(100);
	Otu00046	0.036	-0.609	0.07	0.03	0.38	0.30	0.67	0.39	Bacteria(100);Proteobacteria(100);Gammaproteobacteria(100);Xanthomonadales(100);Rhodanobacteraceae(100);Rhodanobacteraceae_unclassified(100);
	Otu00051	0.042	-0.592	0.11	0.13	0.34	0.39	0.62	0.32	Bacteria(100);Proteobacteria(100);Gammaproteobacteria(100);Pseudomonadales(100);Pseudomonadaceae(100);Pseudomonas(100);
	Otu00067	0.024	0.644	0.13	0.23	0.25	0.39	0.21	0.30	Bacteria(100);Proteobacteria(100);Alphaproteobacteria(100);Micropepsales(99);Micropepsaceae(99);Micropepsaceae_unclassified(99);
	Otu00077	0.048	0.580	0.34	0.04	0.28	0.26	0.14	0.21	Bacteria(100);Deinococcus-Thermus(100);Deinococci(100);Thermales(100);Thermaceae(100);Thermus(100);
	Otu00082	0.025	-0.641	0.13	0.06	0.24	0.09	0.36	0.09	Bacteria(100);Acidobacteria(100);Holophagae(100);Holophagales(100);Holophagaceae(100);Geothrix(100);
	Otu00084	0.040	-0.597	0.03	0.03	0.22	0.19	0.30	0.16	Bacteria(100);Proteobacteria(100);Alphaproteobacteria(100);Rhodospirillales(100);Reyranellaceae(100);Reyranella(100);
	Otu00097	0.037	0.606	0.02	0.09	0.24	0.19	0.12	0.18	Bacteria(100);Actinobacteria(100);Actinobacteria(100);Streptomycetales(100);Streptomycetaceae(100);Kitasatospora(79);

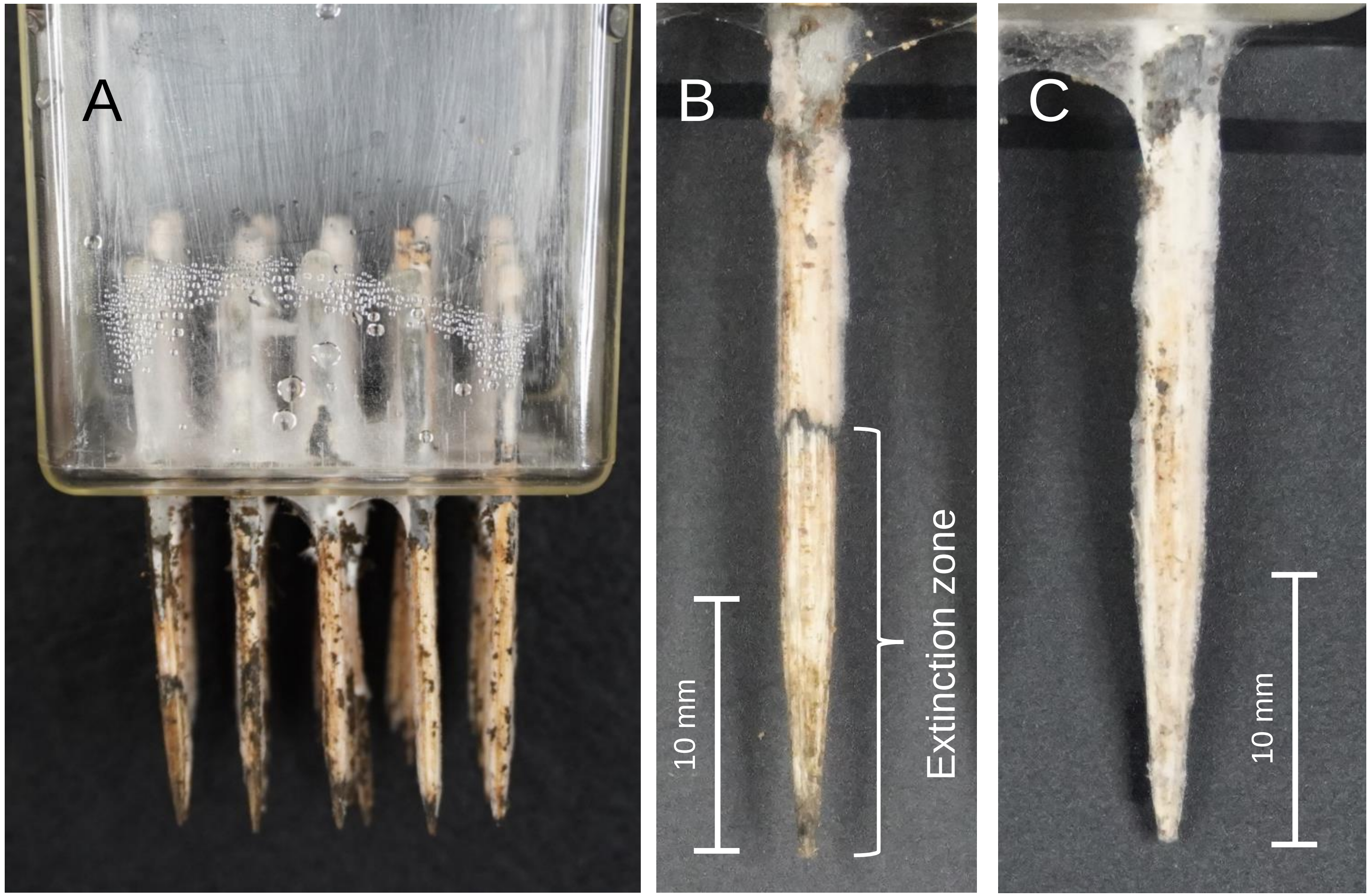

Supplementary Fig. S1 The toothpick method in practice. A, A device used to evaluate soil antagonism against *Rosellinia necatrix*. B and C, Enlargement of toothpick at time of evaluation. In the B, the brace shows the extinction zone of *R. necatrix* on the toothpick. In the C, no extinction zone of *R. necatrix* on the toothpick.

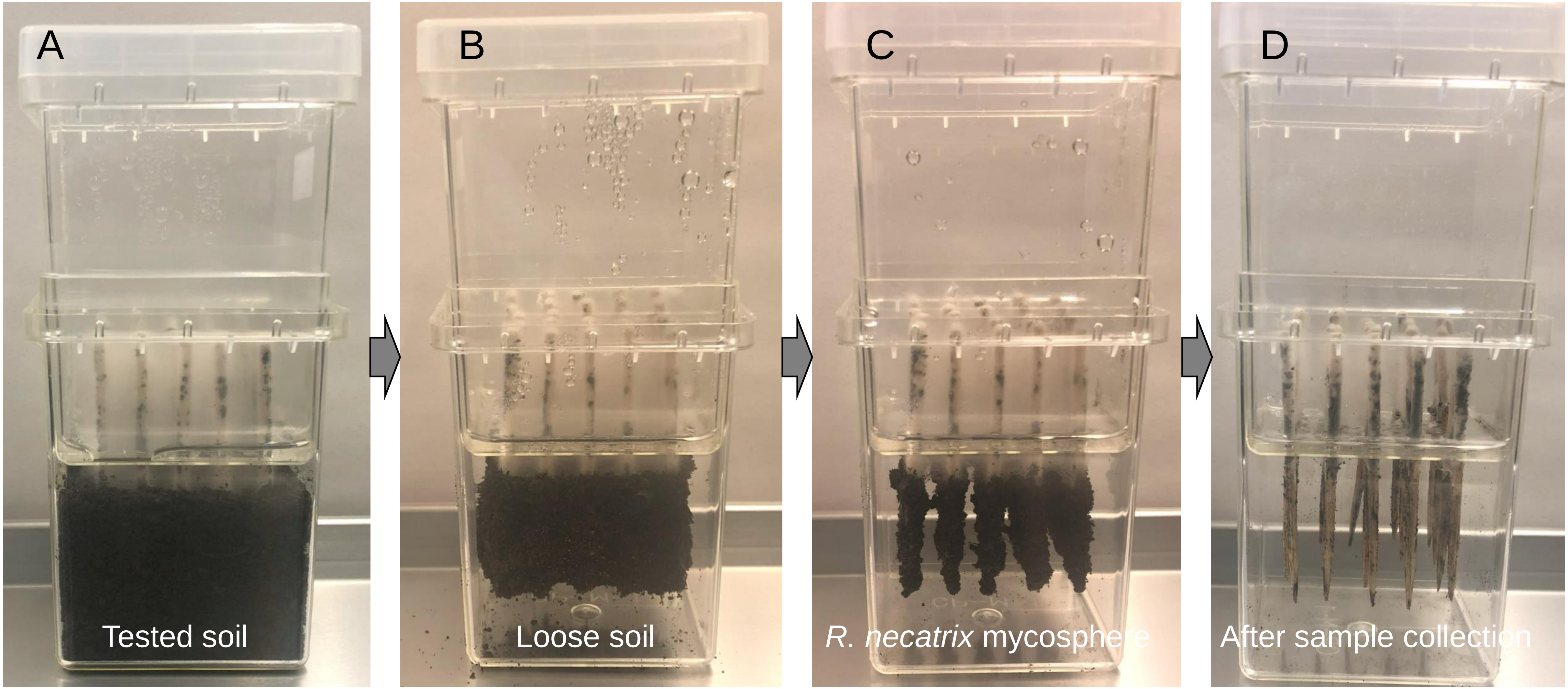

Supplementary Fig. S2 Sample collection of *Rosellinia necatrix* mycosphere from the device used to estimate the soil antagonism against *R. necatrix*. A, A device after incubation with the tested soil. B, Recovered mycelial toothpicks with loose soil particles. C, Soil particles adhered tightly on the mycelial toothpicks, after removing the loose soil particles by shaking. These soil particles were recovered as the *R. necatrix* mycosphere. D, The mycelial toothpicks after stripping off the adhered soil particles.

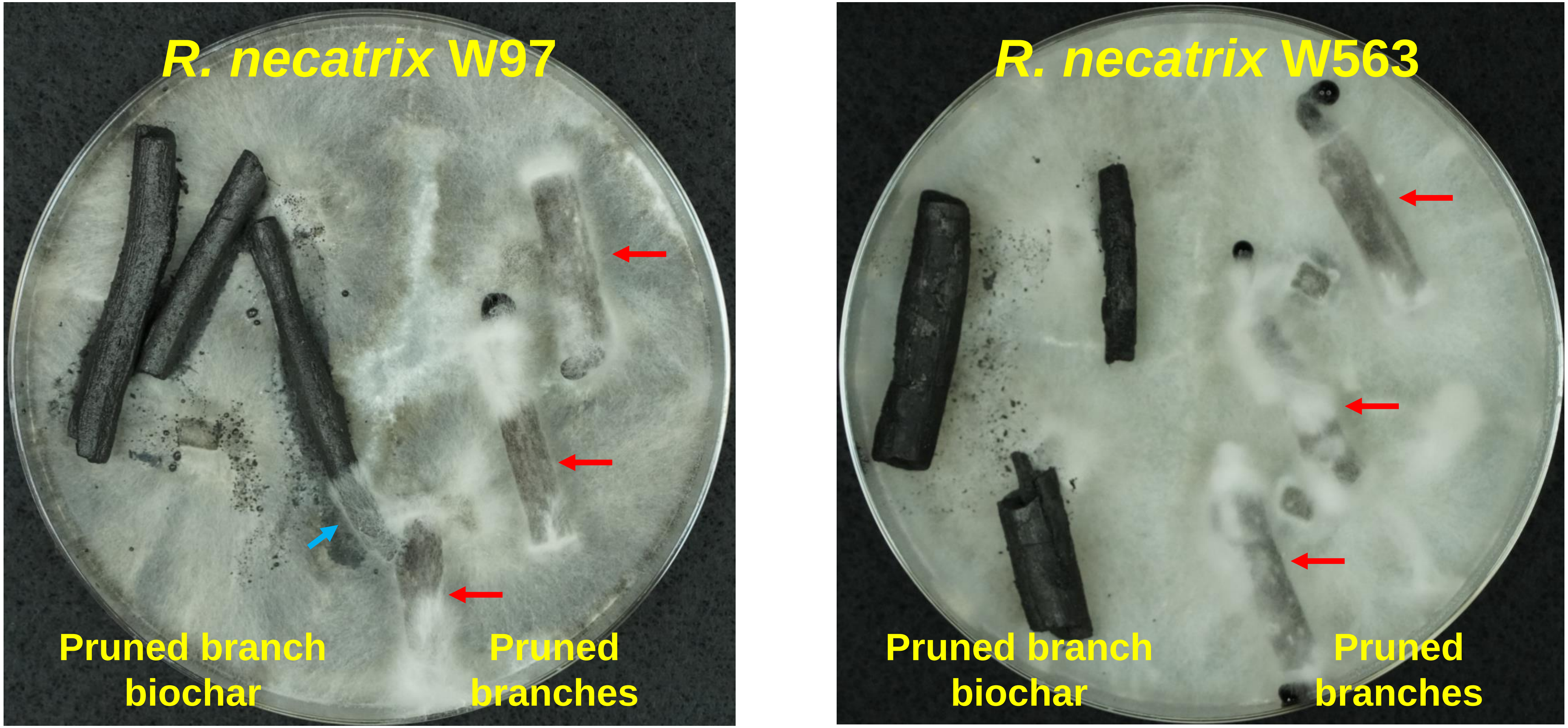

Supplementary Fig. S3 Comparison of the growth of *R. necatrix* on the pruned pear branch (3 pieces on the right side of each photo) and branch biochar (3 pieces on the left side of each photo). The pruned branches and biochar of the pruned branches were autoclaved and placed on the PDA plates with 7 days growing *R. necatrix* strains W97 and W563. Then, the plates were incubated at 23 °C for 7 days to check the mycelial growth of *R. necatrix* on the pruned branch biochar. Red arrows indicate the abundant mycelia growing only on the pruned branches. A blue arrow indicates the W97 mycelia on the biochar, but these originated from the mycelia grown on an adjoin pruned branch.
